# Personalized Boolean network dynamic profiling and machine learning approaches to identify combinatorial treatments for prostate cancer

**DOI:** 10.3389/fphar.2026.1869953

**Published:** 2026-09-29

**Authors:** Se Jin Kim, Sandra Salib, Barbara A. Foster, Anna Woloszynska, Donald E. Mager

**Affiliations:** 1 Division of Pharmacokinetics-Pharmacodynamics & Systems Pharmacology, Department of Pharmaceutical Sciences, University at Buffalo, Buffalo, NY, United States; 2 Department of Pharmacology & Therapeutics, Roswell Park Comprehensive Cancer Center, Buffalo, NY, United States; 3 Enhanced Pharmacodynamics, LLC, Buffalo, NY, United States

**Keywords:** Boolean network modeling, drug combination chemotherapy, machine learning, prostate cancer, quantitative systems pharmacology (QSP)

## Abstract

**Introduction:**

Prostate cancer is the most commonly diagnosed non-cutaneous cancer in males and the second leading cause of male cancer death in the United States. Androgen deprivation therapy to block androgen receptor (AR) signaling is part of the standard of care. However, approximately 30%–40% of patients experience biochemical recurrence (BCR) after primary therapy and progress to a more lethal form of castration-resistant prostate cancer for which there currently is no curative therapy. This study focused on patients at risk of progressing to BCR with the aim of identifying novel combinatorial targets that can be used with AR inhibition to provide greater therapeutic response.

**Methods:**

Personalized Boolean network models were developed using The Cancer Genome Atlas and in-house data to integrate mutations, copy number alterations, and RNA-seq data. Asynchronous stochastic network simulations in MaBoSS (Markovian Boolean Stochastic Simulator) were used to identify potential drug targets in combination with AR inhibition, which were cross-checked using a machine learning algorithm and tested with a cytotoxicity assay across 4 prostate cancer cell lines.

**Results:**

*In silico* predictions and *in vitro* experiments showed that target-specific co-inhibition of E2F1 or Cyclin B with an AR antagonist enhances enzalutamide-associated growth inhibition in all 4 prostate cancer cell lines. Pharmacodynamic modeling suggested that the drug combinations were synergistic with all interaction parameters estimated to be less than 1 (range = 0.156-0.834).

**Discussion:**

Dynamic profiling of personalized Boolean networks in treatment-naïve prostate cancer subjects with and without BCR identified potential drug targets in combination with AR antagonists. Future xenograft studies are needed to confirm *in vitro* results, and exposure-response modeling could provide guidance for feasibility assessment and clinical trial design.

## 1 Introduction

Prostate cancer (PrCa) is the most commonly diagnosed non-cutaneous solid tumor in males and the second leading cause of male cancer death in the United States (American Cancer Society, 2024b). Androgen receptor (AR) and its androgen-dependent (testosterone or dihydrotestosterone) signaling is central to normal prostate development, normal prostate physiology and PrCa progression (Heinlein and Chang, 2004; Eder et al., 2002). A main standard of care for localized disease involves radical prostatectomy or radiation therapy. In contrast, advanced, recurrent, or metastatic PrCa is managed by down regulating AR activity through chemical or surgical castration or administration of antiandrogens such as enzalutamide. Prostate-specific antigen (PSA) is a protein produced primarily by the prostate gland, which can be measured in the blood, and monitoring its concentration provides valuable information about disease progression, treatment response, and PrCa recurrence (Etzioni et al., 2008). Approximately 30%–40% of patients who are initially responsive to therapy based on a decreasing PSA concentration experience biochemical recurrence (BCR), which is characterized by an increase in patient PSA concentrations post-treatment (Freedland et al., 2005). Once patients experience BCR, defined as an increasing PSA following primary therapy, the disease progresses into a more advanced and lethal form of cancer such as castration resistant prostate cancer (CRPC) in which tumor evolves into an androgen independent form that currently lacks durable and curative therapeutic options (Watson et al., 2015).

The use of combination therapies to improve efficacy, limit toxicity, and circumvent tumor resistance has been a central tenet of medical oncology (DeVita and Chu, 2008). The current strategy to combine targeted agents is based on molecular reasoning about the functions of targets: it involves maximizing the inhibition of specific molecules or pathways by targeting multiple components and expanding the inhibition of multiple cellular mechanisms (Dancey and Chen, 2006). However, modeling suggests that some approved and effective combinations appear to provide therapeutic benefit simply owing to patient-to-patient variability and independent drug action rather than drug additivity or synergy (Palmer and Sorger, 2017). This highlights the need to develop a workflow to prioritize the most promising combinations among the large number of available molecular targeted agents for further investigation.

Quantitative systems pharmacology (QSP) integrates the fundamental interactions in biological systems and pharmacology to evaluate drug effects within the context of the properties of the system (Knight-Schrijver et al., 2016; Peter Sorger et al., 2011). QSP employs an integrative approach to connect network-based systems modeling that is complemented with omics data and basic pharmacokinetic and pharmacodynamic principles to enable researchers to make inferences on novel drug targets and combination therapies (Mager and Kimko, 2016; Hart and Xie, 2016). Logic-based models have already shown advantages in successfully describing the dynamics of cellular signal transduction and their dysregulation in cancer (Fumia and Martins, 2013; Montagud et al., 2022; Hu et al., 2015; Saez-Rodriguez et al., 2007; Saadatpour et al., 2011; Chudasama et al., 2015; Saez-Rodriguez et al., 2011; Kirouac et al., 2013). Boolean models are the simplest implementation form of logical models that are more abstract than quantitative models with ordinary differential equations in which the biological components such as genes or proteins are represented as nodes and the regulatory relationship between the nodes are described as edges, which are encoded with logic statements (Kauffman, 1969). Boolean network models that qualitatively describe the complex behavior of signal transduction and regulatory processes (Bloomingdale et al., 2018) that can capture system dynamics in a parsimonious way have been widely utilized to provide insights into combinatorial cancer therapies (Montagud et al., 2022; Eduati et al., 2017; Eduati et al., 2020; Lu et al., 2015).

Although cancer is mainly caused by mutations in cancer-susceptible genes that normally function to maintain cellular genomic stability, cancer progression is a dynamic process (Michor et al., 2004). Specifically, the dynamics of key signaling pathways are crucial in determining cell fate and influencing cancer progression (Purvis and Lahav, 2013; Cohen et al., 2008). For example, a dynamic mathematical model of PrCa that considered major dysregulated signaling pathways (i.e., AR signaling and cell cycle) was constructed and analyzed to further evaluate the mechanisms of tumorigenesis and prioritize drug targets (Montagud et al., 2022). Moreover, investigating dynamic behavior may provide additional complementary insights into the complex underlying biological progression identified from static molecular studies. For example, simulated patient-specific *JNK* pathway dynamics in neuroblastoma patients were able to better correlate with high-risk disease than static *MYCN* amplification (Fey et al., 2015). In Boolean networks, an attractor is a set of long-term asymptotic states that other states evolve towards and can provide insights into the stable configuration of the system or the cellular phenotype (Huang et al., 2005). Often, the asymptotic stable states are studied to test system dynamics, but the transient dynamics towards the attractors are neglected. Investigating the dynamics is particularly relevant in PrCa as AR inhibition results in transient shifts in disease dysregulation (Holzbeierlein et al., 2004).

In this study, the dynamics of patient-specific PrCa Boolean network models constructed from The Cancer Genome Atlas Program (2006) and a Roswell Park Comprehensive Cancer Center (RPCCC) (Ramakrishnan et al., 2024) patient cohort were analyzed to identify combinatorial targets that might provide greater cytotoxicity than AR inhibition alone by screening for target pairs that result in reduced *Proliferation* node activity *in silico* and *in vitro* experimental validation. Machine learning algorithms were applied to dynamic profiles to complement the bioinformatic analyses of static expression data, and experimental validations were performed on four PrCa cell lines to confirm model predictions. The overall study workflow is summarized in Figure 1.

**FIGURE 1:**
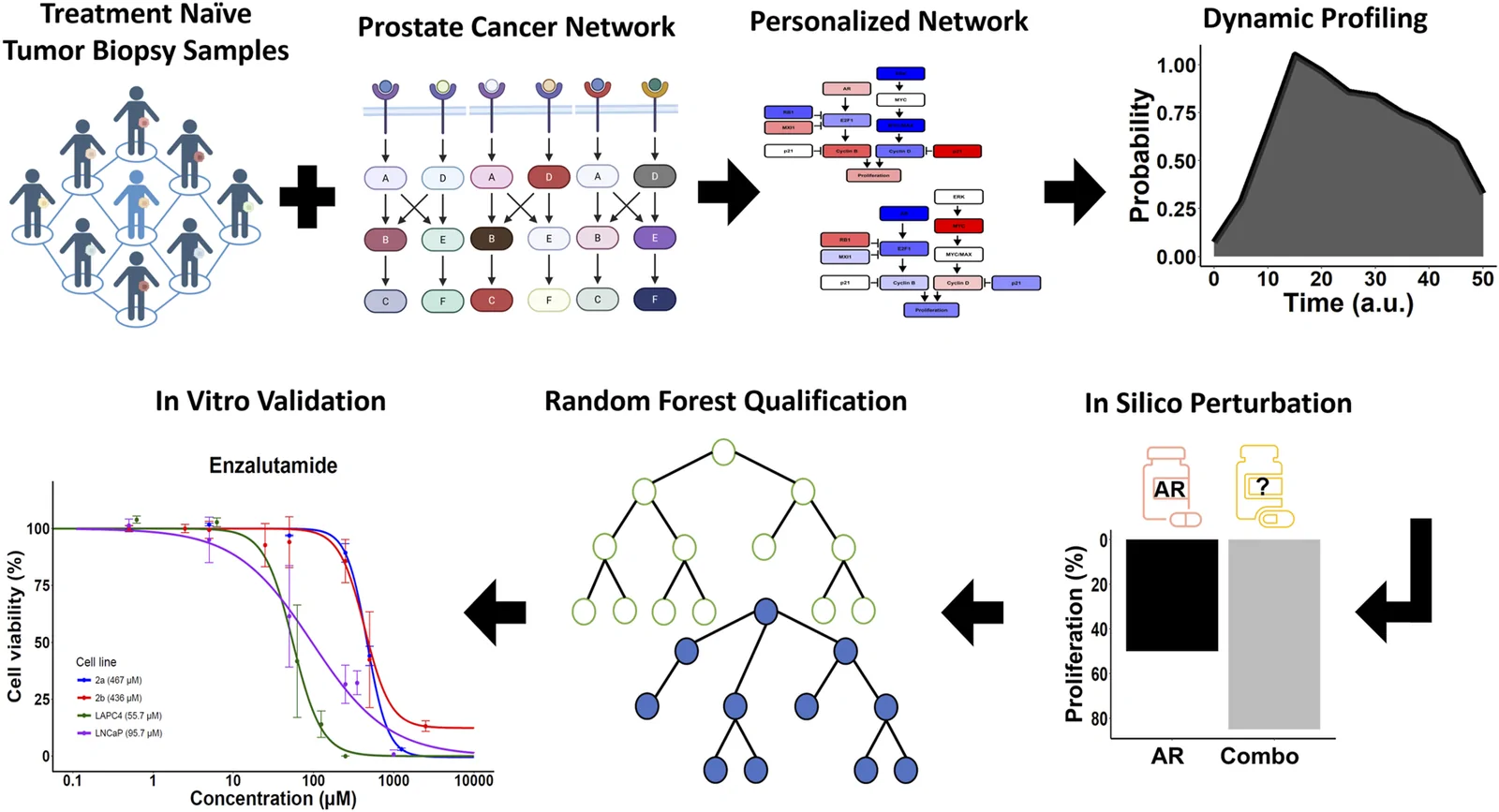
Personalized systems pharmacology workflow for model-guided combination target discovery treatment-naïve prostate tumor biopsy data (i.e., gene mutation, copy number alteration, and RNA sequencing) were integrated into a prior PrCa signaling network (Montagud et al., 2022) to construct patient-specific Boolean models. Stochastic simulations generated dynamic node activity profiles, and *in silico* perturbation was performed to identify AR-inhibition based combinatorial targets. The dynamic profiles were also used as input features for random forest qualification of potential targets. Candidate combination targets were subsequently evaluated through *in vitro* validation experiments in PrCa cell lines.

## 2 Materials and methods

### 2.1 Construction of patient-specific prostate cancer Boolean network models

A prior PrCa Boolean signaling network model with relevant signaling pathways for PrCa (e.g., AKT-PI3K, AR, and ERK pathways among others) was used as a platform to create patient-specific models from patient tumor data (Montagud et al., 2022). The network is comprised of 133 nodes representing a gene or protein that is associated with a discrete level of activity (0 or 1) at each arbitrary unit of time and 499 edges representing a positive or negative regulatory interaction between the source and target nodes (Wang et al., 2012). This network structure was previously subjected to extensive structural validation and sensitivity analyses during its development, which demonstrated that the principal model phenotypic predictions were robust to network perturbations (Montagud et al., 2022). Therefore, the published network was used as a fixed framework for patient-specific personalization.

Patient-specificity was defined by model parameters obtained from mutations, copy number alterations (CNA), and RNA sequencing data using the PROFILE modeling framework (Beal et al., 2018). This approach is an add-on to a logical model simulation software package named Markovian Boolean Stochastic Simulator (MaBoSS) that applies the Gillespie algorithm to the state transition graph, which is defined by a vector of nodes, and performs stochastic simulations to produce temporal trajectories for given sets of initial conditions and state transition rates (activation rate and deactivation rate) (Stoll et al., 2012). PROFILE interprets genomic data, such as mutations and CNA, as binary with an activity node value of 0 or 1 and ignores the logical rules associated with it. Continuous data, such as transcriptomic or proteomic data, are normalized between 0 and 1 to modify the initial condition or state transition rates of a node. This normalization maps relative expression differences into node activity propensities rather than directly representing biochemical activity and does not explicitly infer gene-specific activation thresholds. Nonlinear system-level behavior arises primarily from the Boolean network structure and stochastic state-transition dynamics. This framework was applied to 15 tumor biopsied samples from the RPCCC and 432 samples from TCGA.

All subjects shared the same signaling network that was constructed based on a logic formalism, but each subject has a unique set of parameters derived from their tumor-specific information. In contrast to cross-sectional assessments, MaBoSS simulates the node activation probability over an arbitrary unit of time in a biologically relevant asynchronous manner and thereby reveals the dynamic behavior of signaling pathways and cellular phenotypes. The resulting node trajectories (genes or cellular functions) shows how the system evolves in time under unperturbed or altered conditions (e.g., specific node inhibition or stimulation). Simulating nodal dynamics until steady-state is achieved can identify system attractors. However, we hypothesize that the temporal profiles, or the paths traced between the start and end of the simulation, contain relevant information. Therefore, the area under the curve (AUC) for each node trajectory was calculated and can be interpreted as the net signal flux within that node, which may be tested for associations with phenotypic outputs under different conditions. This allows for a simple representation of a complex system to simulate the evolution of model state probabilities for each patient and investigation of the effects of internal and external perturbations.

### 2.2 *In silico* combinatorial target identification

For each patient-specific model, pharmacological inhibition was simulated by constraining the activity of a given node to a fixed value of zero. A systematic evaluation of 116 pairwise inhibitory combinations with AR antagonism was then performed to identify candidate targets that resulted in greater cytotoxic effects (i.e., reduction in *Proliferation* AUC) relative to the suppression of AR signaling alone. The models were simulated to steady-state to quantify the activity of the nodes over an arbitrary unit of time. For each patient, the simulated trajectories of individual nodes were integrated to compute the node AUC (Equation 1) as a surrogate measure of their dynamic profile:

$${\left[AUC\right]}_{0-t_{\text{ss}}}=\sum_{0}^{t_{ss}}\left(\frac{\left({nodeactivity}_{t1}+{nodeactivity}_{t2}\right)}{2}\times \left(t_{2}-t_{1}\right)\right)$$ (1)

The perturbation effects were assessed by calculating the percent changes in node AUC according to Equation 2 with *bp* and *ap* subscripts denoting before and after perturbation:

$$\Delta AUC\left(\%\right)=\frac{{AUC}_{ap}-{AUC}_{bp}}{{AUC}_{bp}}\times 100$$ (2)

Model codes simulating patient-specific models with inhibitory drug effect are provided in Supplementary Material.

### 2.3 Random forest important feature selection

Random forest is a machine learning algorithm that belongs to the family of ensemble learning methods and used for both classification and regression tasks (Breiman, 2001). It is an aggregate of decision trees that are trained on different training sets that are generated from sampling with replacement of the population and allows for the identification of key features that significantly contribute to the predictive power of a model. The simulated steady-state activation probability, the AUC of each node, and the mRNA expression of the genes for each node were used as separate inputs to predict the BCR phenotype. The models were implemented under the *tidymodels* framework in R version 4.3.3 (Kuhn M, 2020).

The dataset included 432 patients (BCR, n = 81; non-BCR, n = 351) was 80:20 split into a training and test set. Data preprocessing involved removing near zero variance features and normalizing the values between 0 and 1. Principal component analysis was performed on the two data sets to ensure they occupy the same space (Supplementary Figure S1). To improve generalizability and predictive performance, 10-fold cross-validation of samples were repeated 5 times. Grid search parameter tuning was conducted to systematically explore the hyperparameters *mtry*, *min_n*, and *trees*, through a predefined set of hyperparameter values [1,10], [1,15], [800, 1800], to find the combination that yielded the best model performance based of ROC- AUC. *mtry* is number of predictors that will be randomly sampled at each split when creating the tree models, *min_n* is the minimum number of data points in a node that are required for the node to be further split, and *trees* is the number of trees that are contained in the ensemble. Results of the hyperparameter tuning search space are shown in Supplementary Figure S2.

### 2.4 Cell culture

LNCaP and LAPC4 cells were cultured in RPMI-1640 medium (Corning, Corning, NY, United States) supplemented with 10% fetal bovine serum (FBS) (Gibco, Thermo Fisher Scientific, Waltham, MA, United States) and 1% penicillin/streptomycin (P/S) (Corning, Corning, NY, United States). LAPC4 cells were cultured in Poly-D-Lysine Hydrobromide-coated flasks (MP Biomedicals, Irvine, CA, United States). MDA-PCa-2a and MDA-PCa-2b cells were cultured in FNC-coated flasks (AthenaES, Baltimore, MD, United States) with HPC1 medium (AthenaES, Baltimore, MD, United States) supplemented with 20% FBS, 1% P/S, and 1% gentamycin. All cells were maintained at 37 °C and 5% CO2.

### 2.5 Cell cytotoxicity assay

The targets identified through dynamic profiling of the personalized models and important feature analysis of the random forest classification algorithms were used to conduct combinatorial cytotoxic assays with enzalutamide (MedChemExpress, Monmouth Junction, NJ, United States) at various concentrations near the estimated IC_50_. All cells were seeded in a 96 well plate in three replicates overnight accordingly to reach 70%–80% confluency at the time of drug treatment (10,000 cells/well: LNCaP; 20,000 cells/well: LAPC4, MDA-PCA-2a, MDA-PCa-2b). The cells were exposed to vehicle (control), enzalutamide, HLM006474 (MedChemExpress, Monmouth Junction, NJ, United States), or CDK1-IN-2 (MedChemExpress, Monmouth Junction, NJ, United States) for 48 h.

For combinations, the cells were seeded in nine replicates and exposed to nine concentration combinations ranging around single agent IC_50_ values. Cell viability was measured using WST-1 reagent assay (MilliporeSigma, Burlington, MA, United States) at the end of single and combination treatment periods. WST-1 was allowed to incubate for 3 h, and absorbance was measured at 450 nm with a reference wavelength of 690 nm using a SpectraMax 190 microplate reader (Molecular Devices, San Jose, CA, United States).

### 2.6 *In vitro* pharmacodynamic modeling

All *in vitro* PD modeling was conducted using a naïve pooled approach with the maximum likelihood estimation algorithm in *Ubiquity* (Harrold and Abraham, 2014). The drug concentration (*C*) and cell viability (*E*) relationship for each individual drug was characterized with an inhibitory Hill equation according to Equation 3:

$$E=E_{0}.\left[1-\frac{I_{\max}\cdot C^{\gamma }}{{IC}_{50}^{\gamma }+C^{\gamma }}\right]$$ (3)

with *E*_*0*_ as the baseline cell viability, *I*_*max*_ is the maximum inhibition at high concentrations, *IC*_*50*_ is drug concentration producing 50% of *I*_*max*_, and γ is the Hill coefficient.

To investigate the combination effects, single agent parameters (*IC*_*50*_, *Imax*, *γ*) were fixed to the estimated values of the single profiles, and only the interaction parameter (*ψ*) of the non-competitive interaction equation (Equation 4) was estimated, in which ψ < 1, ψ > 1, and ψ = 1 suggests synergistic, antagonistic, and additive interaction (Chakraborty and Jusko, 2002; Niu et al., 2019).

$$f_{u,1+2}=\left(1-\frac{I_{\max,1}\times {\left(\frac{C_{1}}{IC_{50,1}}\right)}^{\gamma 1}}{{\left(\frac{C_{1}}{IC_{50,1}}\right)}^{\gamma 1}+1}\right)\cdot \left(1-\frac{I_{\max,2}\times {\left(\frac{C_{2}}{IC_{50,2}\cdot \psi }\right)}^{\gamma 2}}{{\left(\frac{C_{2}}{IC_{50,2}\cdot \psi }\right)}^{\gamma 2}+1}\right)$$ (4)

*f*_*u*_ is the normalized cell viability after drug exposure, numbered subscripts refer to drug 1 or 2, and ψ is the interaction parameter.

The variance model was defined as Y_obs_ = (σ_1_ + σ _2_ x Y_pred_)^2^, with Y_obs_ as the observed value, Y_pred_ is the model-predicted value, and σ_i_ are the estimated variance parameters. Model evaluation and selection was guided by goodness of fit criteria such as Akaike information criteria, precision and confidence interval of the estimated parameters, examination of residuals, and visual inspection of the model fits.

### 2.7 CDK1 siRNA knock down

SiRNA knock down (KD) of CDK1 was conducted to demonstrate target-specific effects. MDA-PCa-2a and MDA-PCa-2b cells were seeded at 20,000 cells per well in 96 well plates overnight in three replicates. Cells were incubated with vehicle (lipofectamine) (Thermo Fisher Scientific, Waltham, MA, United States) and 10 nM of CDK1 siRNA (Thermo Fisher Scientific, Waltham, MA, United States) for 24 h and washed. 48 h post-siRNA exposure, the cells were incubated at various concentrations of enzalutamide for an additional 48 h, and cell viability was measured as previously described.

### 2.8 Western blot analysis

MDA-PCa-2a cells were seeded in a 6-well plate at a density of 1.0 × 10^6^ cells/well in 1 mL of culture media overnight and treated with vehicle (lipofectamine), 10 nM of scrambled siRNA (Thermo Fisher Scientific, Waltham, MA, United States), or 10 nM of CDK1 siRNA diluted in Opti-MEM (Thermo Fisher Scientific, Waltham, MA, United States) for 24 h, which achieves a persistent protein knockdown for up to 7 days (Thermo Fisher Scientific, 2026). Cells were then washed with PBS and cultured in complete growth media for an additional 48 h and lysed on ice in PierceTM RIPA lysis buffer (Thermo Fisher Scientific, Waltham, MA, United States) supplemented with 1% Halt™ protease inhibitor Cocktail (Thermo Fisher Scientific, Waltham, MA, United States). Samples were centrifuged at 14,000 rpm for 15 min at 4 °C and stored at −20 °C until analysis. Total protein concentration of each sample was quantified by following the Pierce™ Dilution-Free™ Rapid Gold BCA Protein Assay (Thermo Fisher Scientific, Waltham, MA, United States) protocol, and equal amounts (25 μg) of protein for each sample were electrophoresed, separated on SDS-PAGE gel (Bio-Rad, Hercules, CA, United States), and dry transferred to a PVDF membrane (Bio-Rad, Hercules, CA, United States). The membrane was blocked with 5% non-fat dry milk in TSB-T (Tris-buffered saline + 0.1% Tween 20) for 1 h in room temperature and then washed three times with TSB-T for 5 min each and incubated with primary monoclonal antibodies (anti-CDK1, 1:1000, anti-GAPDH, 1:5000) (Pleasanton, CA, United States) for 1 h in room temperature. The membrane was then washed three times with TSB-T for 5 min each and incubated with horseradish peroxidase-conjugated goat anti-rabbit (1:2000) for an hour. After wash, the protein bands were detected with enhanced chemiluminescence solution (Pleasanton, CA, United States) and relatively quantified using Image Lab (Bio-Rad, Hercules, CA, United States).

### 2.9 Statistical analysis

Mann-Whitney U test was used to compare statistical significance in Δ AUC of simulated drug effects. P-value<0.05 was considered statistically significant.

## 3 Results

### 3.1 Investigation of the dynamics of personalized PrCa signaling network models

Patient-specific models were generated for 432 subjects in the TCGA database using a prior PrCa network (Montagud et al., 2022), which captures the signaling pathways and genes relevant for PrCa progression, and stochastic simulations with the Markovian Boolean Stochastic Simulator (MaBoSS) (see Methods section). The Markov Chain simulations on the state transition graph (STG) produce time trajectories to compute and simulate the evolution of model state probabilities (Figures 2A,B). Although patients may have similar node activity at the asymptotic state, the node trajectory among patients can be highly variable (Figure 2C). The AUC of each node was calculated from network-generated trajectories, up until the asymptotic state was reached, to capture the transient dynamics of each node towards its attractor. Patients with similar node activation probabilities (∼0.5) can exhibit different signaling trajectories (Figure 2C), resulting in differences in AUC values with a median of 24.9 and a range from 20.6 to 28.9. These findings demonstrate that dynamic network behavior may contain information that is not captured by steady-state activation probabilities. The percent change in the AUC before and after perturbations for all nodes was subsequently calculated to investigate the systemic effects of drug perturbations (see Methods section).

**FIGURE 2:**
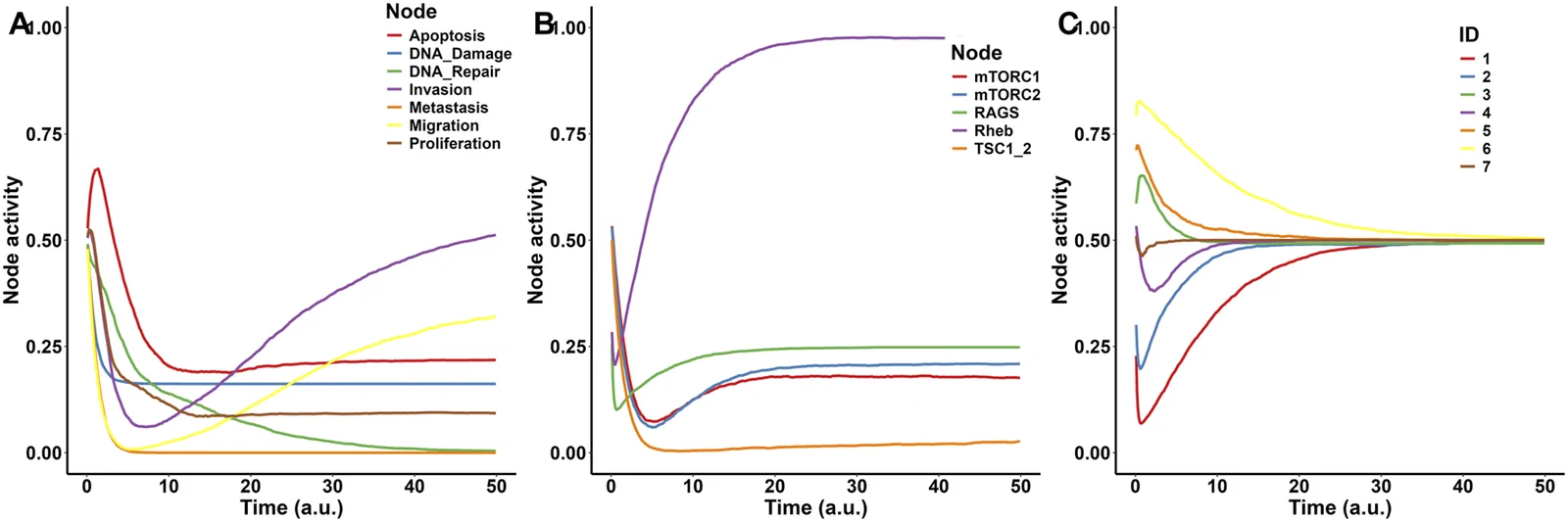
Representative Prostate Cancer Boolean Network Model Simulations. **(A)** Time-course simulation of outcome nodes from a randomly selected patient-specific model generated using the personalized Boolean network framework and simulated in MaBoSS. Node activation probabilities are shown over an arbitrary unit of time. **(B)** Simulation of the mTOR pathway of a randomly selected patient-specific network model, highlighting pathway-specific activation dynamics. **(C)** Hypothetical activity of a single node showing that the dynamic area under the curve (AUC) among patients could vary despite reaching a similar steady-state activation probability. These simulations showcase how an integrated AUC captures dynamic signaling behavior that is not reflected by steady-state measurements alone.

### 3.2 Differences in response to *in silico* AR inhibition by BCR status

Each TCGA patient-specific model was perturbed to mimic AR inhibitor effects by fixing the AR node activity to a discrete value of 0, and the changes in the *Proliferation* AUC were calculated with a mean decrease of 34.8% (SD = 24.3%) across all patients (Supplementary Figure S3). There was a statistically significant difference (P-value < 0.05) in which BCR patients were less responsive to therapy with a mean decrease of 27.8% (SD = 25.0%) in comparison to non-BCR patients with a mean decrease of 36.4% (SD = 24.0%) (Figure 3A). The simulated time trajectories of the *Proliferation* node of three selected patients in each group are shown in Figure 3B, and the greater change in ΔAUC is evident in non-BCR patients. These findings suggest that tumors from patients who experienced BCR might be less dependent on AR signaling and might exhibit reduced sensitivity to AR-targeted therapy.

**FIGURE 3:**
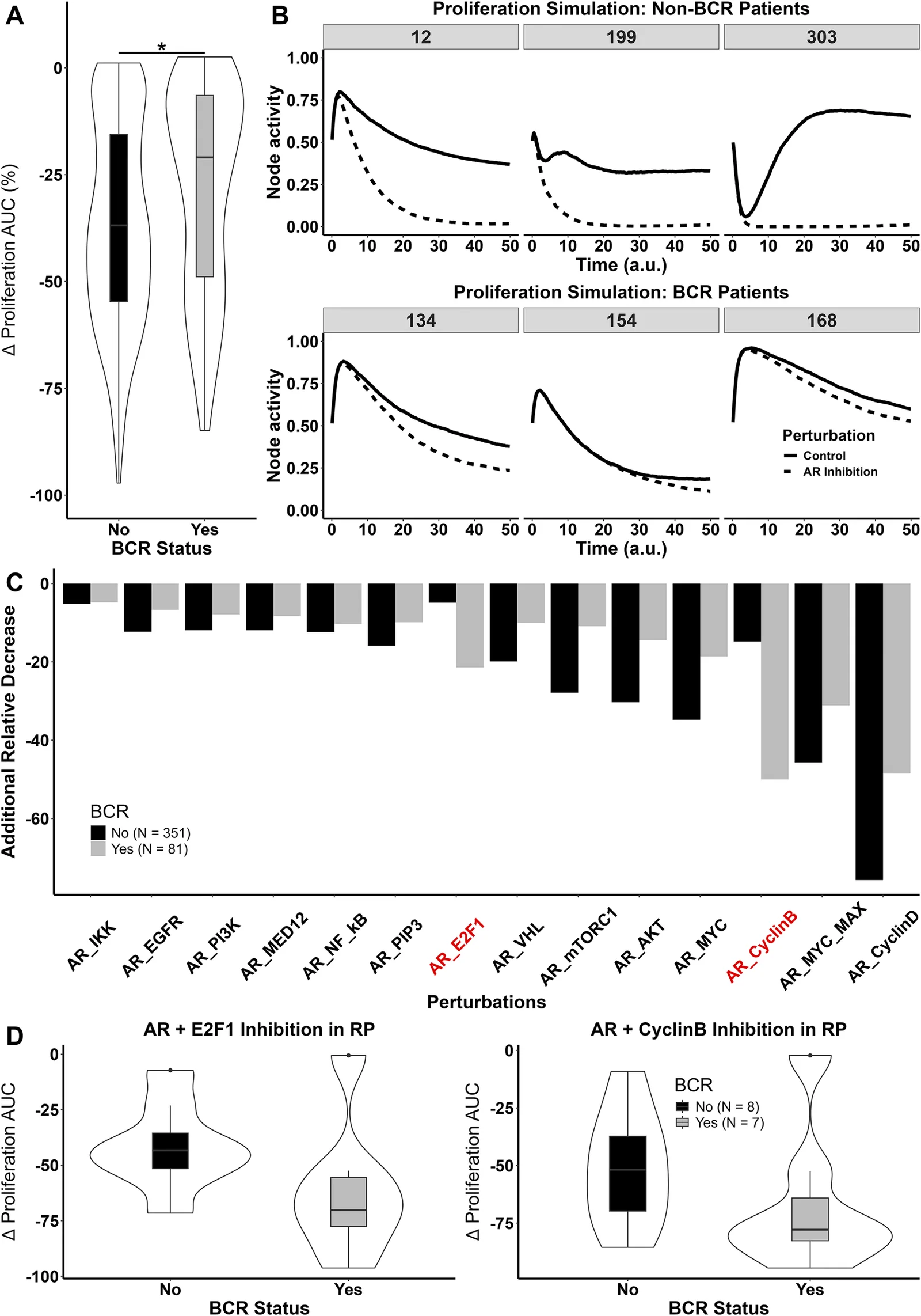
Simulated Drug Response Differences Between BCR and Non-BCR Groups Across Therapeutic Scenarios. **(A)** Significant simulated changes in *Proliferation* node AUC before and after AR inhibition between patients who experienced and did not experience BCR (P-value < 0.05). Model simulations predict that BCR patients may exhibit less changes in *Proliferation* AUC following AR inhibition. **(B)** Representative simulations from three BCR and non-BCR patients illustrating their *Proliferation* node trajectory before and after AR inhibition. Solid line is the model simulation of the baseline condition without AR inhibition, and the dotted line is the model simulation with AR inhibition. Non-BCR patients showed a greater decrease in *Proliferation* AUC after simulated treatment with an AR antagonist. **(C)** Comparisons of additional model simulated decreases in *Proliferation* node AUC after the combinatorial inhibition of AR and targets shown on the x-axis relative to BCR status. Co-inhibition of AR and E2F1 or Cyclin B (highlighted in red) resulted in a greater decrease in *Proliferation* node AUC among BCR-patients. **(D)** Qualification through model simulations of dual inhibition of the predicted targets in the RPCCC cohort (n = 15). Although not statistically significant, simulated co-inhibition of AR and E2F1 or Cyclin B resulted in a greater decrease in *Proliferation* node AUC in BCR-patients.

### 3.3 BCR patient-specific *in silico* combinatorial target identification

Because BCR patients showed a reduced simulated response to AR inhibition, pairwise combinatorial analyses were performed to identify candidate dual targets that might enhance treatment efficacy. The percent change in the *Proliferation* node AUC across 116 pairwise inhibitory combinations with an AR inhibitor were calculated by fixing the discrete value of the two nodes to 0. Of which, 14 combinatorial targets resulted in statistically significant (P-value < 0.05) greater cancer cell killing than AR inhibition alone (Supplementary Figure S4). The extent of additional cell kill of the combinatorial inhibition was further explored by patient BCR status to minimize drug burden and possible adverse effects of additional pharmacological agents on the elderly PrCa patient population. Among the significant combinatorial targets, inhibition of E2F1 or Cyclin B resulted in greater relative reduction in the *Proliferation* node AUC compared to AR inhibition alone in BCR patients when given in combination with an AR inhibitor (Figure 3C).

An external dataset from the RPCCC was used to create 15 patient-specific PrCa Boolean network signaling models to simulate the effects of the dual inhibition of E2F1 or Cyclin B and AR by following the same workflow as previously described (see Methods section). Similar to the model predictions with the TCGA dataset, BCR patients were predicted to show a greater extent of reduced *Proliferation* node AUC in comparison to the non-BCR patients with a median decrease of 61.5% vs. 40.0% after E2F1 and AR inhibition and a median decrease of 71.2% vs. 63.2% after Cyclin B and AR inhibition (Figure 3D). These differences were not statistically significant owing to the relatively small number of subjects. Nevertheless, the consistent direction of the effect observed in the two cohorts provides support for E2F1 and Cyclin B as possible combination targets to enhance the efficacy of AR inhibition therapy in patients at greater risk of tumor recurrence.

A simplified subnetwork of the key AR and ERK/MYC pathways are shown in Figure 4 and provides a mechanistic rationale for the predicted benefits of combining AR inhibition with E2F1 or Cyclin B inhibition. AR signaling promotes the E2F1-Cyclin B axis, whereas ERK/MYC signaling promotes Cyclin D activity, and both Cyclins B/D activate the *Proliferation* node. Cyclin D is also a direct activator of *Proliferation* in the network but was not prioritized in the BCR-specific combination targets, which suggests that the E2F1 and Cyclin B signals reflect patient-specific dependence on the AR-associated cell-cycle axis rather than a consequence of inhibiting any node immediately upstream of *Proliferation*.

**FIGURE 4:**
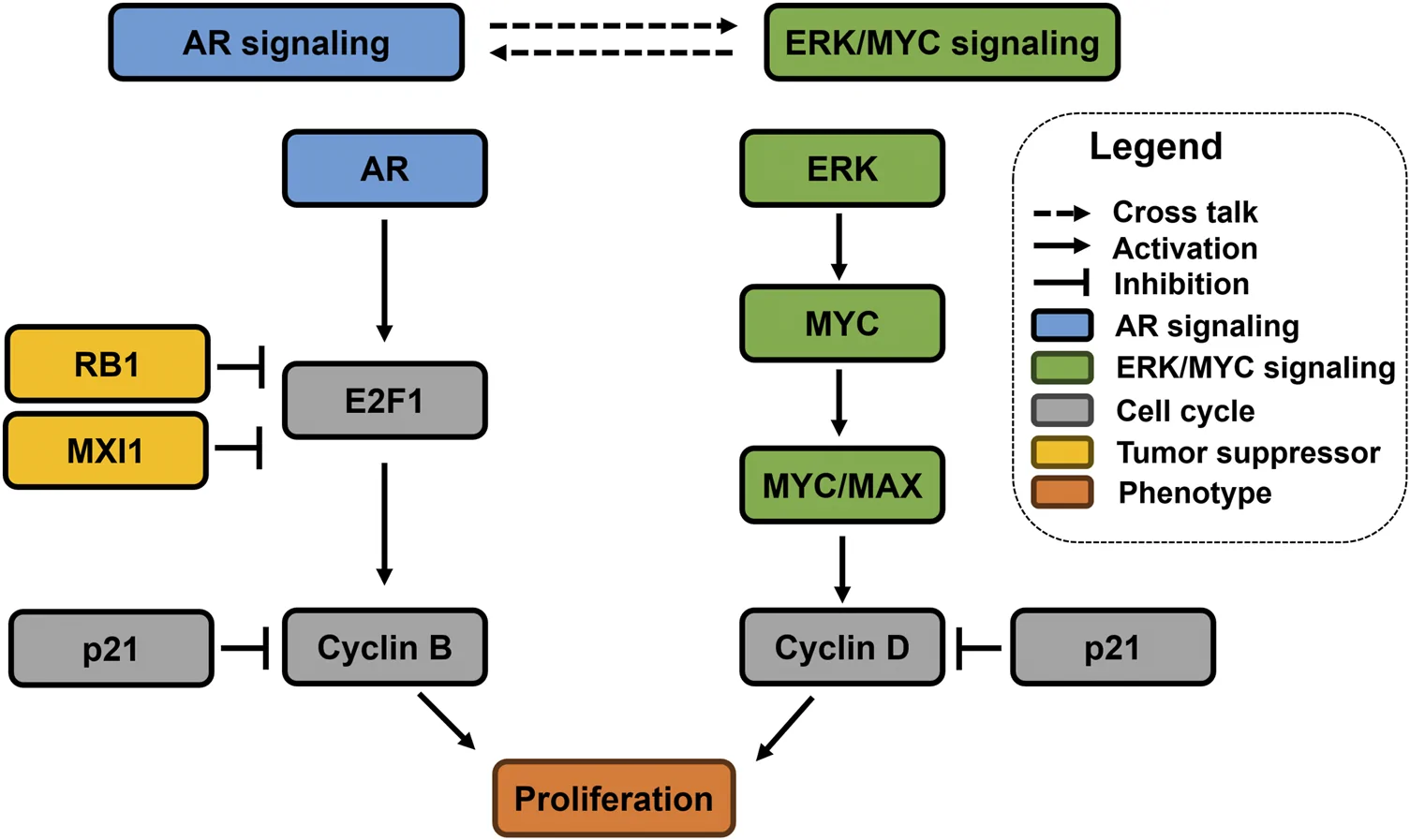
AR and ERK/MYC Subnetwork A focused network was derived from the original prostate cancer Boolean signaling network structure (Montagud et al., 2022) to provide a mechanistic rationale for the model-predicted benefit of combining AR inhibition with E2F1 or Cyclin B inhibition. The subnetwork highlights interactions between AR and ERK/MYC signaling, cell-cycle regulation, and the *Proliferation* node. Key nodes are grouped by their respective colors. Solid arrows indicate activating interactions, arrows with ending blocks indicate inhibitory interactions, and dashed arrows indicate indirect cross-talk between AR and ERK/MYC signaling motifs.

### 3.4 Comparison of dynamic signaling profiles and a machine learning approach

A machine learning algorithm was applied as a complementary analysis to investigate the dynamic profiles of the personalized signaling network models compared to the node activity at the asymptotic state and static mRNA expression from biopsied tumors. Random forest models were fitted with the patient D’Amico Risk Scores, tumor mRNA expression data, simulated steady-state node activity, and calculated AUCs from the simulations of the unperturbed models to classify BCR and non-BCR patients. The D’Amico Risk Score is commonly used in clinical practice to guide treatment options by assessing the risk of PrCa recurrence and progression after treatment, but the ability of this system to provide information for predicting outcomes among individuals has been challenged (D'Amico et al., 1998; Mitchell et al., 2005). The process of model optimization is detailed in Methods.

In the training set, the four levels of data performed similarly in classifying the BCR and non-BCR patients with an AUC of the receiver operating characteristic (ROC) curve of 0.678, 0.695, 0.710, and 0.664 for the steady-state activity, simulated AUC, mRNA expression, and D’Amico Risk Score (Figure 5A). In the test set, simulated AUCs and mRNA expression had similar performances of 0.748 and 0.742, whereas the steady-state activity and D’Amico Risk Score lagged with an AUC of 0.687 and 0.626.

**FIGURE 5:**
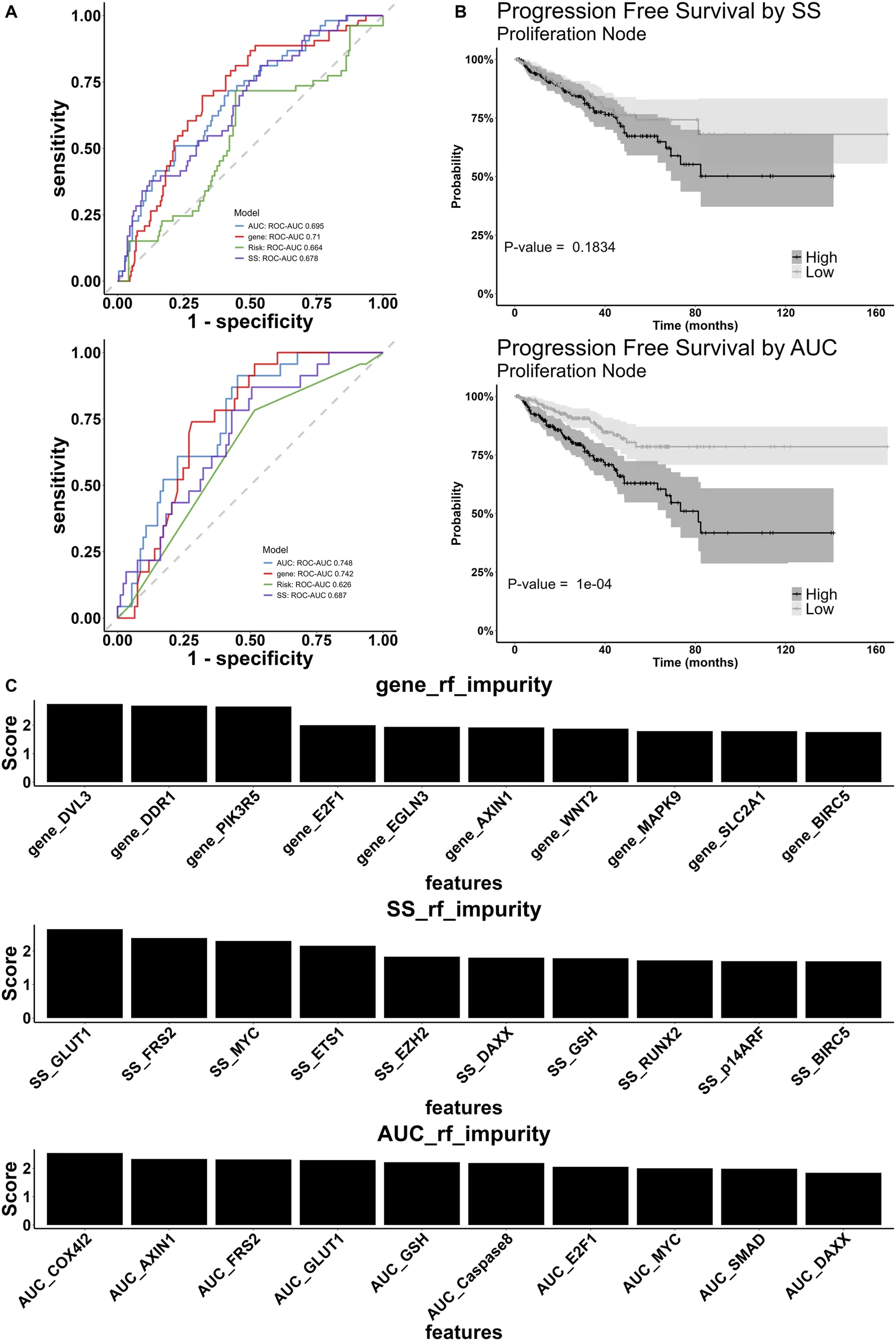
Dynamic and Static Network Descriptors as Predictors of BCR and PFS. **(A)** Comparison of receiver operating characteristic (ROC) curves to evaluate the predictive performance of BCR classification random forest models constructed using different data inputs (blue: *Proliferation* node AUC, red: mRNA expression, green: D’Amico Risk Score, purple: *Proliferation* node steady-state activation probability). ROC curves are shown for the training (upper panel) and test (lower panel) datasets, and the area under the ROC curve values suggest that the models based on *Proliferation* node AUC and mRNA expression can similarly classify BCR subjects. **(B)** Progression-free survival (PFS) stratified based on *Proliferation* node steady-state activation (upper panel) and *Proliferation* node AUC (lower panel). Solid lines are the median survival, and bands are the 95% confidence interval. Dynamic AUC-based stratification provided improved separation of patient outcomes as compared with steady-state activation probability. **(C)** Top 10 highest score features after variable important analysis of each random forest model with different data inputs (upper panel: mRNA expression, middle panel: *Proliferation* node steady-state activation probability, lower panel: *Proliferation* node AUC).

The medians of simulated AUC and steady-state activity of the *Proliferation* node were obtained to classify each patient as “High” or Low” to assess the prognostic significance of the two variables through log-rank test of the progression-free survival (PFS) data. *Proliferation* AUC was significantly associated with PFS with statistical significance; in contrast, the *Proliferation* steady-state activity did not exhibit a significant association with PFS (Figure 5B).

Variance importance is an analysis technique that is used to identify the most influential features in random forest models that contribute to making accurate predictions (Strobl et al., 2007). Greater values of variable importance correspond to a greater positive contribution to the predictive performance of the model as permuting the variable results in a greater decrease in prediction performance. The top 10 variables with the highest variable important scores were selected across the three random forest models of steady-state, AUC, and mRNA expression (Figure 5C). The mRNA expression and simulated AUC of E2F1 largely contribute to their predictive performance of the model, whereas its steady-state activity does not, which parallels with prior analyses of the dynamic signaling profiles.

### 3.5 *In vitro* validation of the combinatorial targets

The nature of drug-drug-interaction (DDI) between the predicted targets of E2F1 and Cyclin B with AR inhibition were investigated in 4 cell lines (LNCaP, LAPC4, MDA-PCa-2a, and MDA-PCa-2b) by conducting single and combinatorial cytotoxic assays with HLM006474 (E2F inhibitor), CDK1-IN-2 (CDK1 inhibitor), and enzalutamide (AR inhibitor). The IC_50_ values of each drug, which is the concentration at which half of maximum killing is observed across the 4 cell lines, were obtained (Table 1) with the individual fits shown in Figures 6A–C. A range of concentrations were used to investigate the type of interaction through pharmacodynamic modeling and the estimates of the interaction parameter (ψ) are summarized in Table 2 The parameter estimates suggest modest synergistic interactions between the two pairwise combinations across all 4 cell lines, in which the total cell kill by the combination was greater than the sum of cell kill of each individual agent.

**TABLE 1:** Parameter estimates for single agent growth inhibition modeling.

Cell line	Enzalutamide (µM)	HLM006474 (µM)	CDK1-IN-2 (µM)
LNCaP	99.6 (32.2%)	1.45 (5.29%)	1.45 (5.29%)
LAPC4	34.3 (12.2%)	36.6 (5.47%)	0.807 (14.0%)
2a	467 (1.59%)	184 (2.83%)	7.13 (18.0%)
2b	436 (5.21%)	124 (6.47%)	12.2 (5.99%)

CV% is in parentheses.

**FIGURE 6:**
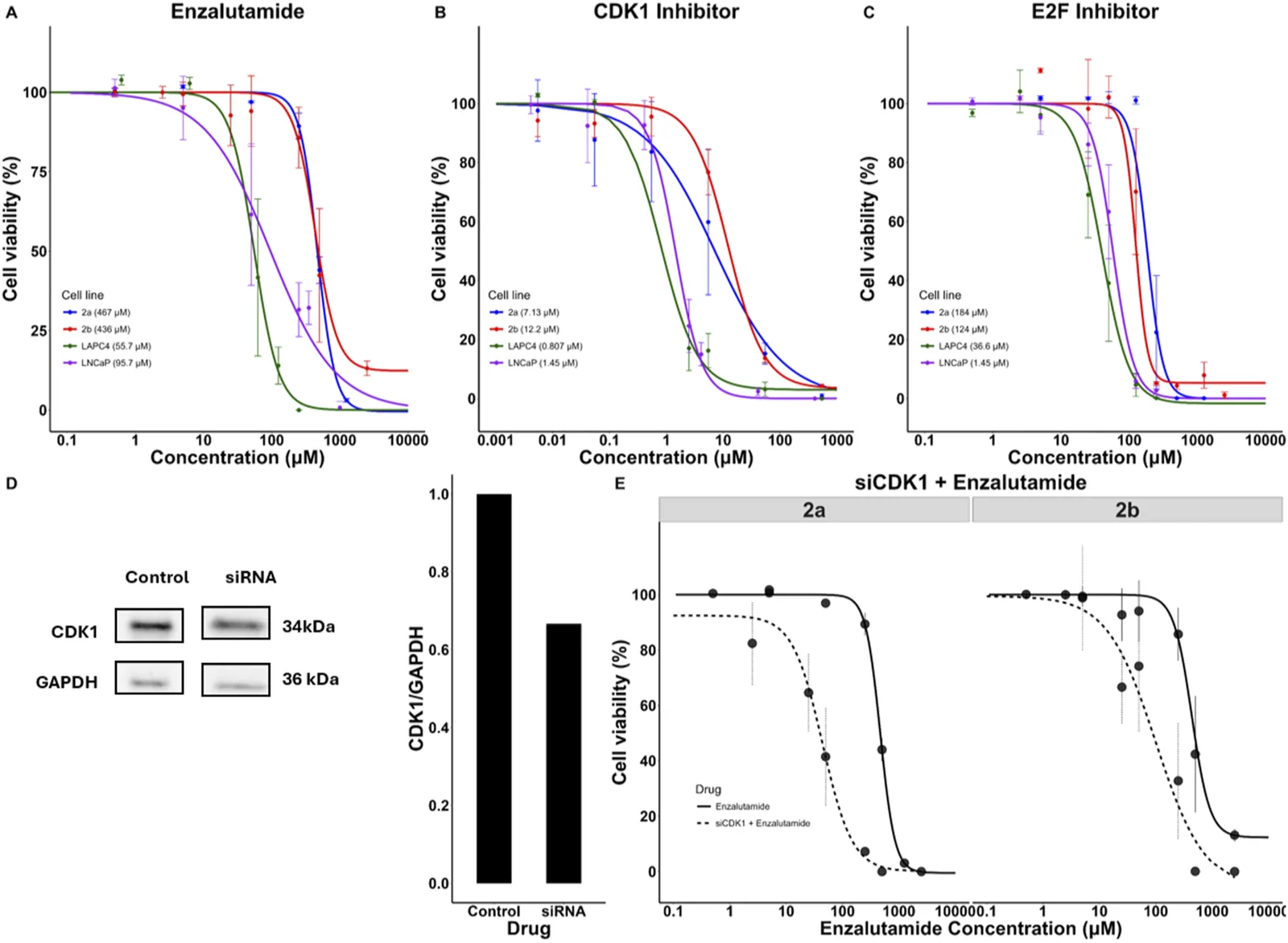
Effect of Individual Drugs and siRNA-mediated Combination on Cell Viability and Drug Sensitivity Across Multiple Cell Lines. **(A–C)** Concentration-cell viability plots for enzalutamide, CDK1-IN-2 (CDK1 inhibitor), and HLM006474 (E2F inhibitor) across four prostate cancer cell lines (blue, MDa-PCa-2a; red, MDA-PCa-2b; green, LAPC4; purple, LNCaP). Cells were treated with increasing concentrations of each inhibitor and viability was measured relative to untreated controls. Mean observed data (dots) and standard deviation (error bars) across triplicates are shown with model-fitted curves (solid lines). **(D)** Western blot analysis of CDK1 and GAPDH in MDa-PCa-2a cells under control conditions and post-siRNA knock down (left panel, 10 nM). Images were digitized and the CDK1/GAPDH relative expression is shown (right panel). **(E)** Concentration-cell viability plots for enzalutamide with (dashed line) and without (solid line) prior CDK1-targeted siRNA exposure in MDa-PCa-2a (left panel) and MDa-PCa-2b (right panel) cell lines. Combined CDK1 suppression and AR inhibition shifted the dose-response curves to the left, which indicated enhanced sensitivity to enzalutamide and suggested that the enhanced efficacy of the enzalutamide with CDK1-IN-2 (Table 2) was CDK1 specific.

**TABLE 2:** Interaction parameter estimates for combination treatments.

Cell line	Enzalutamide + HLM006474	Enzalutamide + CDK1-IN-2
LNCaP	0.156 (6.16%)	0.215 (8.42%)
LAPC4	0.607 (3.40%)	0.834 (3.11%)
2a	0.668 (1.72%)	0.613 (2.91%)
2b	0.439 (4.22%)	0.474 (6.83%)

Estimated interaction parameter (ψ) is unitless, and CV% is in parentheses.

To confirm the target-specific effects of the observed synergy, MDA-PCa-2a and MDA-PCa-2b cell lines were treated with enzalutamide and CDK1 siRNA. Expression of CDK1 was knocked down using siRNA (Figure 6D). The cells were treated with enzalutamide to assess any changes in their cytotoxic profile. MDA-PCA-2a had a 90.6% decrease in its enzalutamide IC_50_ (44.1 µM) and MDA-PCa-2b showed a 77.1% decrease (99.9 µM) (Figure 6E).

## 4 Discussion

PrCa is a heterogenous cancer and characterized by a highly variable disease progression that is dependent on tumor grade and stage at primary diagnosis (Rebello et al., 2021; Haffner et al., 2021). It has a high initial 5-year survival rate of 97% (American Cancer Society, 2024a), but 30%–40% of patients experience BCR, which results in an increased risk of metastatic CRPC for which there is no curative therapy (Rebello et al., 2021; Shore et al., 2024). Approximately 65% of PrCa diagnoses occur in men over 65 years old and 25% over 75 years old (Fitzpatrick, 2008). The age-associated prevalence of the disease underscores the importance of minimizing drug burden and possible additional adverse effects from multiple pharmacological agents.

In this study, personalized systems pharmacology modeling and machine learning approaches were utilized to identify combinatorial targets for PrCa that may achieve greater cancer cell cytotoxicity than the current standard of care of AR inhibition alone, which was tested and confirmed in four PrCa cell lines. Prior network-based approaches have been used to identify drug combinations and evaluate their effects on cancer-associated phenotypes through generic network perturbations (Flobak et al., 2015). The current work builds upon these efforts by incorporating patient-specific molecular data to construct personalized Boolean network models and by evaluating the integrated dynamic response of individual network states. This provides a targeted approach to find effective combinatorial regimens that reduce the dynamic profiles of the *Proliferation* node only in the inherently more aggressive disease in BCR patients. Further research is needed to determine whether reductions in this biomarker is associated with enhanced AR inhibition-mediated reduction in cancer cell growth.

Individual patient data from the TCGA cohort were used to personalize a generic network model of PrCa (Montagud et al., 2022), and temporal patient-specific profiles for each node were generated with MaBoSS under AR inhibition conditions alone or in combination with other node knockouts. Calculated changes in AUC values of the *Proliferation* node provided a quantitative assessment of any enhanced inhibition of proliferation and suggested that co-inhibition of targets in the cell cycle pathway, E2F1 or Cyclin B, in combination with AR inhibition might result in greater anti-cancer effects than AR inhibition alone in BCR patients (Figure 3C). RPCCC patient-specific models showed similar directional trends in which *in silico* co-inhibition of E2F1 and AR or Cyclin B and AR predicted greater reductions in the *Proliferation* node in this subpopulation (Figure 3D). Moreover, co-inhibition of the two combinations through small molecules showed synergistic interactions across all 4 cell lines (Table 2), and the target-specific effects of the observed synergy were supported through siRNA experiments (Figure 6D) and prior studies (Rouaud et al., 2018).

E2F is a family of transcription factors that play a crucial role in the regulation of the cell cycle and cellular proliferation (Johnson and Schneider-Broussard, 1998). The mRNA expression levels of the E2F family are greater in PrCa than in normal prostate tissue, and their elevated expressions are associated with a higher Gleason score, advanced tumor stage, and metastasis (Wang et al., 2021; Han et al., 2022). Survival analysis revealed that E2Fs are significantly associated with worse overall survival and greater risk of BCR (Wang et al., 2021; Han et al., 2022). Cyclin B is a regulatory protein that plays a key role in controlling the cell cycle through the activation of CDK1, which is crucial for initiation and regulation of mitosis (Castedo et al., 2002). In addition, Cyclin B protein expression levels are greater in primary PrCa and CRPC samples than in normal prostate tissue, and its knockdown in CRPC cell lines result in decreased proliferation (Chen et al., 2022). These findings parallel the greater mRNA expression in the BCR patients vs. non-BCR patients within the TCGA cohort (Supplementary Figure S5). Although these relationships are associations rather than causal and increased mRNA expression may not necessarily reflect functional activation, the oncogenic potential of these genes in PrCa reaffirms their selection as possible therapeutic targets.

The machine learning approach in this study showed that investigating the simulated dynamics of a node is non-inferior to its steady-state activity and measured mRNA expression level (Figure 5A). However, in contrast with static expression, this type of abstract framework provides an opportunity to apply computational approaches to predict dynamic behavior of a biological system in response to environmental stimuli such as drug effects and internal perturbations (Saadatpour et al., 2010). Moreover, the dynamic activities were significant classifiers of PFS unlike the *Proliferation* nodal steady-state activities at the network attractor (Figure 5B). A similar approach was conducted in triple-negative breast cancer patients in which the dynamics of the personalized ErbB receptor signaling network were analyzed and compared to the selected gene panels that were used in the model (Imoto et al., 2022). The temporal dynamics of AKT, ERK, and c-Myc were able to accurately predict the differences in prognosis and sensitivity to kinase inhibitors in contrast to just gene expression. Moreover, Fey et al. characterized the c-Jun N-terminal kinase (JNK) pathway in neuroblastoma and uncovered that the dynamics in this pathway contain prognostically relevant information (Fey et al., 2015). The inability to initiate switch-like JNK activation based on patient-specific model simulations was significantly associated with poor overall survival regardless of the static *MYCN* amplification status, which is a major neuroblastoma oncogene (Ora and Eggert, 2011).

The modeling and simulation workflow in this study provides opportunities for biomarker development. The dynamic activity signatures that go beyond static gene expression could serve as network-informed biomarkers to stratify patients based on disease progression or therapy response. This is particularly valuable in PrCa in which traditional biological biomarkers such as PSA face numerous limitations such as poor specificity (Porzycki and Ciszkowicz, 2020; Prensner et al., 2012). Computational biomarkers derived from personalized models that are constructed from patient-specific molecular data may improve monitoring disease progression and treatment strategies. Because this framework relies on molecular data from routinely collected biopsied tumors, it could potentially be incorporated into clinical workflows to support patient stratification, prioritize combination therapies, and therapeutic monitoring for further evaluations or interventions.

In addition to clinical decision making, this modeling framework has the potential to be applied in the future across diverse types of cancer such as breast cancer, which is also characterized by a high recurrence rate. Synergistic combination therapy has been shown to help prevent tumor recurrences in breast cancer tumor-bearing mice and significantly reduce recurrences or death from any causes by approximately 37% (Sun et al., 2019; Schmid et al., 2020). The presented methodology (Figure 1) utilizes readily available data such as genomics and transcriptomics as compared with proteomic data (Sidoli et al., 2017), and the integration of machine learning enhances its scalability and robustness by confirming the identification of important combinatorial targets within the complex and dynamic signaling landscape to facilitate the practice of precision medicine.

There are several limitations to this study. It is recognized that the final network could be biased towards the TCGA data as the base PrCa Boolean signaling network, initially constructed from a generic cancer Boolean model (Fumia and Martins, 2013), was extended to include prostate cancer-specific genes and pathways using TCGA transcriptomic data. However, the *in vitro* experiments across four PrCa cell lines confirmed model combinatorial predictions of increased cytotoxicity. The semi-qualitative nature of this logical modeling approach limits the findings only to the discovery of potential targets, and it does not provide quantitative dose-exposure-response relationships needed to determine optimal dosing regimens. The construction of quantitative models such as ODE-based deterministic systems that are fitted to dynamic data can provide such information. Furthermore, although the *in vitro* validation experiments in this study confirmed the synergistic interaction, limited translation from *in vitro* to *in vivo* models for cancer require further evaluation in preclinical *in vivo* models (Hum et al., 2020; Poondru et al., 2002).

In summary, this study identified combinatorial targets that provide greater antiproliferative effects than AR inhibition alone by analyzing the signaling dynamics of personalized PrCa Boolean network models that are complemented by a machine learning approach and validated with *in vitro* experiments. These findings have real-world applications as AR inhibition is routinely used to treat advanced PrCa and further investigation is needed to confirm these findings in preclinical models and characterize the exposure-response relationships.

## Data Availability

Publicly available datasets were analyzed in this study. This data can be found here: TCGA (as mentioned in the article) and NCBI, with accession numbers GSE237995, GSE262522, and GSE262524.
