# Supplementary material for: Personalized Boolean network dynamic profiling and machine learning approaches to identify combinatorial treatments for prostate cancer
Source: Front Pharmacol. 2026 Sep 29;17:1869953. doi: 10.3389/fphar.2026.1869953 (PMC13658504; doi:10.3389/fphar.2026.1869953)
Supplement: Supplementary file 1 [file Supplementaryfile1.docx]

**Personalized Boolean Network Dynamic Profiling and Machine Learning Approaches to Identify Combinatorial Treatments for Prostate Cancer**

Supplementary Material

Figure S1. Principal Component Analysis between Training and Test Sets

Principal component analysis was conducted on both training and test sets of the random forest models, which were constructed with different data inputs (left panel: mRNA expression, middle panel: steady-state activation probability, right panel: simulated AUC) after an 80:20 split. For each data input, the training and test set occupy the same space.

Figure S2. Hyperparameter Tuning of each Random Forest Model

Grid search parameter tuning was conducted on three hyperparameters: *mtry*, *min_n*, and *number of trees*. For each model, a set of hyperparameters that resulted in the smallest ROC AUC was selected. Minimal number of data points in a node (*min_n*) and number of trees at the predefined ranges had limited effect on ROC AUC for all models. ROC AUC increased with greater number of randomly selected predictors (*mtry*) for mRNA expression (upper panel), whereas ROC AUC decreased with greater *mtry* for steady-state activation probability (middle panel) and simulated AUC (lower panel).

Figure S3. Distribution of Percent Change in *Proliferation* node AUC after AR Inhibition

Percent change in *Proliferation* node AUC before and after AR inhibition for each patient in the TCGA cohort (n = 432).

Figure S4. Combinatorial Targets Resulting in Significant Differences in $\boldsymbol{\Delta}$ AUC of *Proliferation* in comparison to AR inhibition alone

116 pairwise inhibitory combinations with AR antagonism were screened to identify targets that result in greater cytotoxicity than AR inhibition alone. 14 targets resulted in a significantly greater decrease in *Proliferation* node AUC. The median percent change in AUC across the 432 patients is highlighted underneath each bar.

Figure S5. E2F1 and Cyclin B mRNA Expression in TCGA cohort

Comparison of E2F1 and Cyclin B (CCNB1) mRNA expression between patients who experienced BCR vs. patients who did not experience BCR. mRNA expression of both genes was significantly greater (P-value < 0.05) in BCR patients (grey) vs. non-BCR patients (black).
