# Supplementary material for: Personalized Boolean network dynamic profiling and machine learning approaches to identify combinatorial treatments for prostate cancer
Source: Front Pharmacol. 2026 Sep 29;17:1869953. doi: 10.3389/fphar.2026.1869953 (PMC13658504; doi:10.3389/fphar.2026.1869953)
Supplement: Supplementary file 2 [file Supplementaryfile2.docx]

**Personalized Boolean Network Dynamic Profiling and Machine Learning Approaches to Identify Combinatorial Treatments for Prostate Cancer**

Supplementary Material

**Instructions**

- There are three sections to this document: one python script to run simulations (Pages 2-6), one .bnd file that contains the model structure with logic gates (Pages 7-24), and one .cfg file that defines the parameter values (Pages 24-36). .bnd and .cfg files come directly from the Supplementary materials of Montagud et al., eLife, 2022.
- Save the .bnd and .cfg files as separate, independent .txt files and save as .bnd and .cfg.
- Set paths in the python script to appropriate directory

**Simulate patient-specific models**

"""

Run MaBoSS simulations for one or more Boolean network models.

This script:

1. Finds .bnd model files in an input directory

2. Matches each .bnd file with its corresponding .cfg file

3. Mutates selected nodes (e.g., forces them OFF)

4. Runs the simulation

5. Saves node probability trajectories to CSV

Requirements:

pip install pandas numpy maboss

Notes:

- Update the USER SETTINGS section below before running.

- This script assumes each .bnd file has a matching .cfg file

with the same base name in the same directory.

"""

from pathlib import Path

import time

import pandas as pd

import maboss

\# =========================

\# USER SETTINGS

\# =========================

\# Directory containing input .bnd and .cfg files

INPUT_DIR = Path("/path/to/your/model_directory")

\# Directory where CSV outputs will be saved

OUTPUT_DIR = Path("/path/to/save/results")

\# Nodes to mutate

NODES_TO_MUTATE = ["AR"]

\# Mutation state: typically "OFF" or "ON"

MUTATION_STATE = "OFF"

\# MaBoSS simulation parameters

MAX_TIME = 50

\# =========================

\# HELPER FUNCTIONS

\# =========================

def load_model(bnd_file: Path, cfg_file: Path, max_time: int):

"""

Load a MaBoSS model and update simulation parameters.

Parameters

----------

bnd_file : Path

Path to the .bnd file.

cfg_file : Path

Path to the .cfg file.

max_time : int

Maximum simulation time.

Returns

-------

model

Loaded MaBoSS model object.

"""

model = maboss.load(str(bnd_file), str(cfg_file))

model.param["max_time"] = max_time

return model

def run_model_with_mutation(bnd_file: Path, cfg_file: Path, node_name: str,

mutation_state: str = "OFF", max_time: int = 50) -> pd.DataFrame:

"""

Load a model, mutate one node, run the simulation, and return node trajectories.

Parameters

----------

bnd_file : Path

Path to the .bnd file.

cfg_file : Path

Path to the .cfg file.

node_name : str

Node to mutate.

mutation_state : str, optional

Mutation state to apply ("OFF" or "ON"), by default "OFF".

max_time : int, optional

Maximum simulation time, by default 50.

Returns

-------

pd.DataFrame

Node probability trajectory table.

"""

model = load_model(bnd_file, cfg_file, max_time=max_time)

model_nodes = list(model.network.keys())

model.network.set_output(model_nodes)

if node_name not in model_nodes:

raise ValueError(

f"Node '{node_name}' not found in model '{bnd_file.name}'. "

f"Available nodes: {model_nodes}"

)

mutated_model = maboss.copy_and_mutate(model, [node_name], mutation_state)

simulation = mutated_model.run()

node_probtraj = simulation.get_nodes_probtraj()

return node_probtraj

def find_model_pairs(input_dir: Path):

"""

Find matching .bnd and .cfg file pairs in a directory.

Parameters

----------

input_dir : Path

Directory containing model files.

Returns

-------

list[tuple[Path, Path]]

List of (bnd_file, cfg_file) pairs.

"""

model_pairs = []

for bnd_file in sorted(input_dir.glob("*.bnd")):

cfg_file = bnd_file.with_suffix(".cfg")

if cfg_file.exists():

model_pairs.append((bnd_file, cfg_file))

else:

print(f"Warning: No matching .cfg file found for {bnd_file.name}; skipping.")

return model_pairs

\# =========================

\# MAIN WORKFLOW

\# =========================

def main():

script_start_time = time.time()

if not INPUT_DIR.exists():

raise FileNotFoundError(f"Input directory does not exist: {INPUT_DIR}")

OUTPUT_DIR.mkdir(parents=True, exist_ok=True)

model_pairs = find_model_pairs(INPUT_DIR)

if not model_pairs:

raise FileNotFoundError(

f"No matching .bnd/.cfg model pairs found in: {INPUT_DIR}"

)

print(f"Input directory: {INPUT_DIR}")

print(f"Output directory: {OUTPUT_DIR}")

print(f"Nodes to mutate: {NODES_TO_MUTATE}")

print(f"Mutation state: {MUTATION_STATE}")

print(f"Max time: {MAX_TIME}")

print(f"Found {len(model_pairs)} model pair(s).\n")

for bnd_file, cfg_file in model_pairs:

print(f"Processing model: {bnd_file.name}")

for node in NODES_TO_MUTATE:

run_start_time = time.time()

try:

node_df = run_model_with_mutation(

bnd_file=bnd_file,

cfg_file=cfg_file,

node_name=node,

mutation_state=MUTATION_STATE,

max_time=MAX_TIME,

)

output_file = OUTPUT_DIR / f"node_{node}_{bnd_file.stem}.csv"

node_df.to_csv(output_file, index=False)

elapsed = time.time() - run_start_time

print(f" Saved: {output_file.name} ({elapsed:.2f} s)")

except Exception as exc:

print(f" Error while processing node '{node}' for {bnd_file.name}: {exc}")

total_elapsed = time.time() - script_start_time

print(f"\nTotal script time: {total_elapsed:.2f} seconds")

if __name__ == "__main__":

main()

**Example .bnd file**

Node Acidosis {

logic = (Acidosis);

rate_up = @logic ? $u_Acidosis : 0;

rate_down = @logic ? 0 : $d_Acidosis;

}

Node AKT {

logic = ((HSPs | (PDK1 & PIP3) | PIP3 | (SHH & PIP3)) & !PTCH1);

rate_up = @logic ? $u_AKT : 0;

rate_down = @logic ? 0 : $d_AKT;

}

Node AMPK {

logic = (ATR | HIF1 | AMP_ATP | ATM) & !FGFR3;

rate_up = @logic ? $u_AMPK : 0;

rate_down = @logic ? 0 : $d_AMPK;

}

Node AMP_ATP {

logic = (!Nutrients);

rate_up = @logic ? $u_AMP_ATP : 0;

rate_down = @logic ? 0 : $d_AMP_ATP;

}

Node Androgen {

logic = (Androgen);

rate_up = @logic ? $u_Androgen : 0;

rate_down = @logic ? 0 : $d_Androgen;

}

Node Angiogenesis {

logic = (VEGF & (NF_kB | SMAD));

rate_up = @logic ? $u_Angiogenesis : 0;

rate_down = @logic ? 0 : $d_Angiogenesis;

}

Node APAF1 {

logic = ((Caspase8 | BAX | p53 | Bak | HSPs) & !Bcl_XL & !BCL2 & !AKT);

rate_up = @logic ? $u_APAF1 : 0;

rate_down = @logic ? 0 : $d_APAF1;

}

Node Apoptosis {

logic = (Caspase9 | Caspase8 | Caspase3);

rate_up = @logic ? $u_Apoptosis : 0;

rate_down = @logic ? 0 : $d_Apoptosis;

}

Node AR {

logic = ((GLI | EP300 | HSPs | NKX3_1 | EZH2 | NCOA3 | PKC | SMAD | Androgen) & !PTEN & !NCOR1 & !NCOR2 & !MDM2);

rate_up = @logic ? $u_AR : 0;

rate_down = @logic ? 0 : $d_AR;

}

Node AR_ERG {

logic = ((AR & fused_event) | (AR & fused_event & !NKX3_1));

rate_up = @logic ? $u_AR_ERG : 0;

rate_down = @logic ? 0 : $d_AR_ERG;

}

Node ATM {

logic = (DNA_Damage);

rate_up = @logic ? $u_ATM : 0;

rate_down = @logic ? 0 : $d_ATM;

}

Node ATR {

logic = (DNA_Damage);

rate_up = @logic ? $u_ATR : 0;

rate_down = @logic ? 0 : $d_ATR;

}

Node AXIN1 {

logic = (GSK3);

rate_up = @logic ? $u_AXIN1 : 0;

rate_down = @logic ? 0 : $d_AXIN1;

}

Node BAD {

logic = (!AKT & !HIF1 & !p90RSK & !RAF & !YWHAZ);

rate_up = @logic ? $u_BAD : 0;

rate_down = @logic ? 0 : $d_BAD;

}

Node Bak {

logic = (Caspase8);

rate_up = @logic ? $u_Bak : 0;

rate_down = @logic ? 0 : $d_Bak;

}

Node BAX {

logic = (!BCL2 & !HIF1 & !YWHAZ & (JNK | HIF1 | p53) & !ETS1);

rate_up = @logic ? $u_BAX : 0;

rate_down = @logic ? 0 : $d_BAX;

}

Node BCL2 {

logic = ((HSPs | NF_kB | RUNX2) & !BAX & !p53 & !DAXX & !BAD);

rate_up = @logic ? $u_BCL2 : 0;

rate_down = @logic ? 0 : $d_BCL2;

}

Node Bcl_XL {

logic = (AR & !BAD & !p53);

rate_up = @logic ? $u_Bcl_XL : 0;

rate_down = @logic ? 0 : $d_Bcl_XL;

}

Node beta_catenin {

logic = (!GSK3 & !p53 & !AXIN1 & (EZH2 | YWHAZ));

rate_up = @logic ? $u_beta_catenin : 0;

rate_down = @logic ? 0 : $d_beta_catenin;

}

Node BIRC5 {

logic = (HSPs & !p53);

rate_up = @logic ? $u_BIRC5 : 0;

rate_down = @logic ? 0 : $d_BIRC5;

}

Node BMP2 {

logic = (GLI & (TGFBR | FGFR3));

rate_up = @logic ? $u_BMP2 : 0;

rate_down = @logic ? 0 : $d_BMP2;

}

Node BRCA1 {

logic = ((E2F1 | ATM | ATR | (E2F1 & EP300) | (ATM & EP300) | (ATR & EP300)) & !CyclinD & !Bak);

rate_up = @logic ? $u_BRCA1 : 0;

rate_down = @logic ? 0 : $d_BRCA1;

}

Node BRCA2 {

logic = (ATM & ATR & EP300);

rate_up = @logic ? $u_BRCA2 : 0;

rate_down = @logic ? 0 : $d_BRCA2;

}

Node Carcinogen {

logic = (Carcinogen);

rate_up = @logic ? $u_Carcinogen : 0;

rate_down = @logic ? 0 : $d_Carcinogen;

}

Node Caspase3 {

logic = (Caspase8 | Caspase9);

rate_up = @logic ? $u_Caspase3 : 0;

rate_down = @logic ? 0 : $d_Caspase3;

}

Node Caspase8 {

logic = (!cFLAR & FADD);

rate_up = @logic ? $u_Caspase8 : 0;

rate_down = @logic ? 0 : $d_Caspase8;

}

Node Caspase9 {

logic = ((!AKT & CytoC & APAF1 & PTCH1) | (!AKT & BIRC5 & CytoC & APAF1) | (!AKT & BIRC5 & CytoC & APAF1 & PTCH1));

rate_up = @logic ? $u_Caspase9 : 0;

rate_down = @logic ? 0 : $d_Caspase9;

}

Node CDH2 {

logic = (TWIST1);

rate_up = @logic ? $u_CDH2 : 0;

rate_down = @logic ? 0 : $d_CDH2;

}

Node cFLAR {

logic = ((AKT | AR) & !JNK);

rate_up = @logic ? $u_cFLAR : 0;

rate_down = @logic ? 0 : $d_cFLAR;

}

Node CHK1_2 {

logic = (ATM & ATR & BRCA1);

rate_up = @logic ? $u_CHK1_2 : 0;

rate_down = @logic ? 0 : $d_CHK1_2;

}

Node COX4I2 {

logic = (HIF1);

rate_up = @logic ? $u_COX4I2 : 0;

rate_down = @logic ? 0 : $d_COX4I2;

}

Node CyclinB {

logic = (E2F1 & !p21);

rate_up = @logic ? $u_CyclinB : 0;

rate_down = @logic ? 0 : $d_CyclinB;

}

Node CyclinD {

logic = ((GLI | TCF | NF_kB) & MYC_MAX & !p21 & !FOXO & !p15 & !GSK3);

rate_up = @logic ? $u_CyclinD : 0;

rate_down = @logic ? 0 : $d_CyclinD;

}

Node CytoC {

logic = ((Caspase8 | BAX | p53 | Bak) & !Bcl_XL & !BCL2 & !AKT);

rate_up = @logic ? $u_CytoC : 0;

rate_down = @logic ? 0 : $d_CytoC;

}

Node DAXX {

logic = (!ATM & !ATR & !SPOP);

rate_up = @logic ? $u_DAXX : 0;

rate_down = @logic ? 0 : $d_DAXX;

}

Node DNA_Damage {

logic = ((Carcinogen | (Carcinogen & ROS)) & !SPOP);

rate_up = @logic ? $u_DNA_Damage : 0;

rate_down = @logic ? 0 : $d_DNA_Damage;

}

Node DNA_Repair {

logic = (ATM & ATR & (BRCA1 | BRCA2));

rate_up = @logic ? $u_DNA_Repair : 0;

rate_down = @logic ? 0 : $d_DNA_Repair;

}

Node Dsh {

logic = (WNT);

rate_up = @logic ? $u_Dsh : 0;

rate_down = @logic ? 0 : $d_Dsh;

}

Node E2F1 {

logic = ((AR | (FOXA1 & AR) | (ATR & AR) | (ATM & AR)) & !RB1 & !MXI1);

rate_up = @logic ? $u_E2F1 : 0;

rate_down = @logic ? 0 : $d_E2F1;

}

Node eEF2 {

logic = (!eEF2K);

rate_up = @logic ? $u_eEF2 : 0;

rate_down = @logic ? 0 : $d_eEF2;

}

Node eEF2K {

logic = (p90RSK | p70S6kab);

rate_up = @logic ? $u_eEF2K : 0;

rate_down = @logic ? 0 : $d_eEF2K;

}

Node EGF {

logic = (EGF);

rate_up = @logic ? $u_EGF : 0;

rate_down = @logic ? 0 : $d_EGF;

}

Node EGFR {

logic = ((!TGFBR & !FRS2 & !EGFR & !PKC & (ERK | Androgen | EGF)) | (!TGFBR & !FRS2 & (Androgen | EGF)));

rate_up = @logic ? $u_EGFR : 0;

rate_down = @logic ? 0 : $d_EGFR;

}

Node EMT {

logic = (CDH2 & !E_cadherin & !FOXA1);

rate_up = @logic ? $u_EMT : 0;

rate_down = @logic ? 0 : $d_EMT;

}

Node EP300 {

logic = (AKT);

rate_up = @logic ? $u_EP300 : 0;

rate_down = @logic ? 0 : $d_EP300;

}

Node ERG {

logic = (ERK);

rate_up = @logic ? $u_ERG : 0;

rate_down = @logic ? 0 : $d_ERG;

}

Node ERK {

logic = (RAF | MEK1_2);

rate_up = @logic ? $u_ERK : 0;

rate_down = @logic ? 0 : $d_ERK;

}

Node ETS1 {

logic = (!p53 & (RTK | ERK));

rate_up = @logic ? $u_ETS1 : 0;

rate_down = @logic ? 0 : $d_ETS1;

}

Node ETV1 {

logic = (ETS1 | MEK1_2);

rate_up = @logic ? $u_ETV1 : 0;

rate_down = @logic ? 0 : $d_ETV1;

}

Node EZH2 {

logic = (AKT & E2F1 & (ERG | AR_ERG));

rate_up = @logic ? $u_EZH2 : 0;

rate_down = @logic ? 0 : $d_EZH2;

}

Node E_cadherin {

logic = ((beta_catenin | AR_ERG) & !Snail & !ERG & !NF_kB & !Slug);

rate_up = @logic ? $u_E_cadherin : 0;

rate_down = @logic ? 0 : $d_E_cadherin;

}

Node FADD {

logic = (PTCH1 | TNFalpha) & !SHH & !MAP3K1_3;

rate_up = @logic ? $u_FADD : 0;

rate_down = @logic ? 0 : $d_FADD;

}

Node FGF {

logic = (FGF);

rate_up = @logic ? $u_FGF : 0;

rate_down = @logic ? 0 : $d_FGF;

}

Node FGFR3 {

logic = ((FGF | (FGF & RUNX2)) & !FRS2 & !PKC & !TGFBR);

rate_up = @logic ? $u_FGFR3 : 0;

rate_down = @logic ? 0 : $d_FGFR3;

}

Node FOS {

logic = (ERK | ETS1 | AR);

rate_up = @logic ? $u_FOS : 0;

rate_down = @logic ? 0 : $d_FOS;

}

Node FOXA1 {

logic = (FOXO);

rate_up = @logic ? $u_FOXA1 : 0;

rate_down = @logic ? 0 : $d_FOXA1;

}

Node FOXO {

logic = (MAP3K1_3 & JNK & !AKT);

rate_up = @logic ? $u_FOXO : 0;

rate_down = @logic ? 0 : $d_FOXO;

}

Node FRS2 {

logic = (!ERK & !EGFR & FGFR3 & !FRS2 & !TGFBR);

rate_up = @logic ? $u_FRS2 : 0;

rate_down = @logic ? 0 : $d_FRS2;

}

Node fused_event {

logic = (fused_event);

rate_up = @logic ? $u_fused_event : 0;

rate_down = @logic ? 0 : $d_fused_event;

}

Node GADD45 {

logic = (SMAD | p53);

rate_up = @logic ? $u_GADD45 : 0;

rate_down = @logic ? 0 : $d_GADD45;

}

Node GLI {

logic = ((WNT | SMO) & !SPOP);

rate_up = @logic ? $u_GLI : 0;

rate_down = @logic ? 0 : $d_GLI;

}

Node GLUT1 {

logic = ((HIF1 | MYC_MAX | AKT) & Nutrients);

rate_up = @logic ? $u_GLUT1 : 0;

rate_down = @logic ? 0 : $d_GLUT1;

}

Node Glycolysis {

logic = (GLUT1 & Lactic_acid);

rate_up = @logic ? $u_Glycolysis : 0;

rate_down = @logic ? 0 : $d_Glycolysis;

}

Node GSH {

logic = (MYC_MAX | NF_kB | p21);

rate_up = @logic ? $u_GSH : 0;

rate_down = @logic ? 0 : $d_GSH;

}

Node GSK3 {

logic = (!AKT & !Dsh & MEK1_2 & !mTORC2 & !p90RSK);

rate_up = @logic ? $u_GSK3 : 0;

rate_down = @logic ? 0 : $d_GSK3;

}

Node HIF1 {

logic = (!PHDs & !VHL & !p53 & !FOXO & (HSPs | mTORC1 | mTORC2 | Hypoxia | MYC_MAX));

rate_up = @logic ? $u_HIF1 : 0;

rate_down = @logic ? 0 : $d_HIF1;

}

Node HSPs {

logic = ((PKC | FOXA1 | Carcinogen) & !BRCA1 & !E2F1 & !AKT);

rate_up = @logic ? $u_HSPs : 0;

rate_down = @logic ? 0 : $d_HSPs;

}

Node Hypermethylation {

logic = (!IDH1);

rate_up = @logic ? $u_Hypermethylation : 0;

rate_down = @logic ? 0 : $d_Hypermethylation;

}

Node Hypoxia {

logic = (Hypoxia);

rate_up = @logic ? $u_Hypoxia : 0;

rate_down = @logic ? 0 : $d_Hypoxia;

}

Node IDH1 {

logic = (SMAD | FOXA1 | mTORC2 | ((SMAD | mTORC2 | FOXA1) & MEK1_2));

rate_up = @logic ? $u_IDH1 : 0;

rate_down = @logic ? 0 : $d_IDH1;

}

Node IKK {

logic = ((mTORC2 | AKT) & (PKC | TAK1 | ETS1) & !PHDs & !p53);

rate_up = @logic ? $u_IKK : 0;

rate_down = @logic ? 0 : $d_IKK;

}

Node Invasion {

logic = ((CDH2 & SMAD) | (CDH2 & SMAD & EZH2) | beta_catenin);

rate_up = @logic ? $u_Invasion : 0;

rate_down = @logic ? 0 : $d_Invasion;

}

Node JNK {

logic = ((ATM | GADD45 | TAK1 | MAP3K1_3) & !ERK & !p38);

rate_up = @logic ? $u_JNK : 0;

rate_down = @logic ? 0 : $d_JNK;

}

Node JUN {

logic = (AR | ETS1 | JNK);

rate_up = @logic ? $u_JUN : 0;

rate_down = @logic ? 0 : $d_JUN;

}

Node Lactic_acid {

logic = (LDHA);

rate_up = @logic ? $u_Lactic_acid : 0;

rate_down = @logic ? 0 : $d_Lactic_acid;

}

Node LDHA {

logic = (HIF1 | MYC_MAX | IDH1);

rate_up = @logic ? $u_LDHA : 0;

rate_down = @logic ? 0 : $d_LDHA;

}

Node MAP3K1_3 {

logic = (RAS & !(FOS | JUN | JNK | p38));

rate_up = @logic ? $u_MAP3K1_3 : 0;

rate_down = @logic ? 0 : $d_MAP3K1_3;

}

Node MDM2 {

logic = (((AKT | p53) & DAXX & !p14ARF & !ATR & !ATM));

rate_up = @logic ? $u_MDM2 : 0;

rate_down = @logic ? 0 : $d_MDM2;

}

Node MED12 {

logic = (!GLI);

rate_up = @logic ? $u_MED12 : 0;

rate_down = @logic ? 0 : $d_MED12;

}

Node MEK1_2 {

logic = (MAP3K1_3 | RAF | PDK1);

rate_up = @logic ? $u_MEK1_2 : 0;

rate_down = @logic ? 0 : $d_MEK1_2;

}

Node Metastasis {

logic = (Migration & AR_ERG);

rate_up = @logic ? $u_Metastasis : 0;

rate_down = @logic ? 0 : $d_Metastasis;

}

Node Migration {

logic = (((AKT & ERK) | (AKT & ETV1)) & Invasion & EMT);

rate_up = @logic ? $u_Migration : 0;

rate_down = @logic ? 0 : $d_Migration;

}

Node mTORC1 {

logic = ((AKT | (AKT & Rheb)) & !AMPK & !TSC1_2);

rate_up = @logic ? $u_mTORC1 : 0;

rate_down = @logic ? 0 : $d_mTORC1;

}

Node mTORC2 {

logic = ((!AMPK & RAGS & Rheb) | (AKT & !AMPK & Rheb) | (AKT & !AMPK & RAGS) | (AKT & RAGS & Rheb & !AMPK));

rate_up = @logic ? $u_mTORC2 : 0;

rate_down = @logic ? 0 : $d_mTORC2;

}

Node MXI1 {

logic = (HIF1);

rate_up = @logic ? $u_MXI1 : 0;

rate_down = @logic ? 0 : $d_MXI1;

}

Node MYC {

logic = ((GLI | ERK | TCF | JUN | FOS | AR_ERG | ERG | NF_kB | SHH) & (mTORC1 | E2F1 | p38) & !HIF1);

rate_up = @logic ? $u_MYC : 0;

rate_down = @logic ? 0 : $d_MYC;

}

Node MYC_MAX {

logic = (MYC & !MXI1 & !TGFBR & !SMAD);

rate_up = @logic ? $u_MYC_MAX : 0;

rate_down = @logic ? 0 : $d_MYC_MAX;

}

Node NCOA3 {

logic = (!SPOP & p38);

rate_up = @logic ? $u_NCOA3 : 0;

rate_down = @logic ? 0 : $d_NCOA3;

}

Node NCOR1 {

logic = (!AKT);

rate_up = @logic ? $u_NCOR1 : 0;

rate_down = @logic ? 0 : $d_NCOR1;

}

Node NCOR2 {

logic = (!AKT & !FOXO);

rate_up = @logic ? $u_NCOR2 : 0;

rate_down = @logic ? 0 : $d_NCOR2;

}

Node NF1 {

logic = (!PKC);

rate_up = @logic ? $u_NF1 : 0;

rate_down = @logic ? 0 : $d_NF1;

}

Node NF_kB {

logic = ((E_cadherin | IKK | DNA_Damage) & (NCOA3 | PIP3 | Snail));

rate_up = @logic ? $u_NF_kB : 0;

rate_down = @logic ? 0 : $d_NF_kB;

}

Node NKX3_1 {

logic = (PKC & AR & !ERG & !AR_ERG);

rate_up = @logic ? $u_NKX3_1 : 0;

rate_down = @logic ? 0 : $d_NKX3_1;

}

Node Nutrients {

logic = (Nutrients);

rate_up = @logic ? $u_Nutrients : 0;

rate_down = @logic ? 0 : $d_Nutrients;

}

Node p14ARF {

logic = (E2F1 & MYC_MAX & RAS);

rate_up = @logic ? $u_p14ARF : 0;

rate_down = @logic ? 0 : $d_p14ARF;

}

Node p15 {

logic = (ZBTB17);

rate_up = @logic ? $u_p15 : 0;

rate_down = @logic ? 0 : $d_p15;

}

Node p21 {

logic = ((p53 | SMAD | HIF1 | ZBTB17) & !TERT & !MYC_MAX & !MDM2 & !AKT & !ERK);

rate_up = @logic ? $u_p21 : 0;

rate_down = @logic ? 0 : $d_p21;

}

Node p38 {

logic = (MAP3K1_3 & !ERK & !GADD45);

rate_up = @logic ? $u_p38 : 0;

rate_down = @logic ? 0 : $d_p38;

}

Node p53 {

logic = ((Acidosis | CHK1_2 | p38 | HIF1) & !BCL2 & !MDM2 & !HSPs & !Snail);

rate_up = @logic ? $u_p53 : 0;

rate_down = @logic ? 0 : $d_p53;

}

Node p70S6kab {

logic = (mTORC2 | PDK1);

rate_up = @logic ? $u_p70S6kab : 0;

rate_down = @logic ? 0 : $d_p70S6kab;

}

Node p90RSK {

logic = (PDK1 | ERK);

rate_up = @logic ? $u_p90RSK : 0;

rate_down = @logic ? 0 : $d_p90RSK;

}

Node PDK1 {

logic = (HIF1 | MYC_MAX | PIP3);

rate_up = @logic ? $u_PDK1 : 0;

rate_down = @logic ? 0 : $d_PDK1;

}

Node PHDs {

logic = (!Hypoxia & ROS);

rate_up = @logic ? $u_PHDs : 0;

rate_down = @logic ? 0 : $d_PHDs;

}

Node PI3K {

logic = (TGFBR | FRS2 | EGFR);

rate_up = @logic ? $u_PI3K : 0;

rate_down = @logic ? 0 : $d_PI3K;

}

Node PIP3 {

logic = ((!p53 & PI3K & !PTEN) | (!p53 & PI3K));

rate_up = @logic ? $u_PIP3 : 0;

rate_down = @logic ? 0 : $d_PIP3;

}

Node PKC {

logic = (RTK & WNT);

rate_up = @logic ? $u_PKC : 0;

rate_down = @logic ? 0 : $d_PKC;

}

Node Proliferation {

logic = (CyclinB | CyclinD);

rate_up = @logic ? $u_Proliferation : 0;

rate_down = @logic ? 0 : $d_Proliferation;

}

Node PTCH1 {

logic = (!SHH & GLI);

rate_up = @logic ? $u_PTCH1 : 0;

rate_down = @logic ? 0 : $d_PTCH1;

}

Node PTEN {

logic = ((!NF_kB & !Snail & FGFR3 & p53) | (!NF_kB & !Snail & p53));

rate_up = @logic ? $u_PTEN : 0;

rate_down = @logic ? 0 : $d_PTEN;

}

Node Quiescence {

logic = (!Apoptosis & !Proliferation);

rate_up = @logic ? $u_Quiescence : 0;

rate_down = @logic ? 0 : $d_Quiescence;

}

Node RAF {

logic = (!EZH2 & RAS) | (EZH2);

rate_up = @logic ? $u_RAF : 0;

rate_down = @logic ? 0 : $d_RAF;

}

Node RAGS {

logic = (!Hypoxia & Nutrients);

rate_up = @logic ? $u_RAGS : 0;

rate_down = @logic ? 0 : $d_RAGS;

}

Node RAS {

logic = (EGFR | TGFBR | FRS2 | FGFR3);

rate_up = @logic ? $u_RAS : 0;

rate_down = @logic ? 0 : $d_RAS;

}

Node RB1 {

logic = (!CyclinB & !CyclinD & CHK1_2 & !p53 & !AMPK);

rate_up = @logic ? $u_RB1 : 0;

rate_down = @logic ? 0 : $d_RB1;

}

Node Rheb {

logic = (!TSC1_2);

rate_up = @logic ? $u_Rheb : 0;

rate_down = @logic ? 0 : $d_Rheb;

}

Node ROS {

logic = ((COX4I2 | Hypoxia) & !GSH);

rate_up = @logic ? $u_ROS : 0;

rate_down = @logic ? 0 : $d_ROS;

}

Node RTK {

logic = (EGFR | FGFR3);

rate_up = @logic ? $u_RTK : 0;

rate_down = @logic ? 0 : $d_RTK;

}

Node RUNX2 {

logic = (!ETS1 & !FOXO & Slug) | (ETS1 & !FOXO);

rate_up = @logic ? $u_RUNX2 : 0;

rate_down = @logic ? 0 : $d_RUNX2;

}

Node SHH {

logic = (!BMP2 & !ATR);

rate_up = @logic ? $u_SHH : 0;

rate_down = @logic ? 0 : $d_SHH;

}

Node Slug {

logic = ((TCF | NF_kB) & !DAXX & !p53 & !MDM2);

rate_up = @logic ? $u_Slug : 0;

rate_down = @logic ? 0 : $d_Slug;

}

Node SMAD {

logic = ((TNFalpha | TGFBR) & !AR);

rate_up = @logic ? $u_SMAD : 0;

rate_down = @logic ? 0 : $d_SMAD;

}

Node SMO {

logic = (!PTCH1 & SHH);

rate_up = @logic ? $u_SMO : 0;

rate_down = @logic ? 0 : $d_SMO;

}

Node Snail {

logic = ((GLI | NF_kB | SMAD | beta_catenin) & !GSK3 & !p53);

rate_up = @logic ? $u_Snail : 0;

rate_down = @logic ? 0 : $d_Snail;

}

Node SPOP {

logic = (SPOP);

rate_up = @logic ? $u_SPOP : 0;

rate_down = @logic ? 0 : $d_SPOP;

}

Node TAK1 {

logic = (TGFBR | TNFalpha);

rate_up = @logic ? $u_TAK1 : 0;

rate_down = @logic ? 0 : $d_TAK1;

}

Node TCF {

logic = ((beta_catenin | AR_ERG | ERG) & !TAK1);

rate_up = @logic ? $u_TCF : 0;

rate_down = @logic ? 0 : $d_TCF;

}

Node TERT {

logic = ((GLI | AKT | HIF1 | MYC_MAX | NF1 | NF_kB | MYC_MAX) & !ZBTB17 & !p53 & !eEF2 & !SMAD);

rate_up = @logic ? $u_TERT : 0;

rate_down = @logic ? 0 : $d_TERT;

}

Node TGFb {

logic = (TGFb);

rate_up = @logic ? $u_TGFb : 0;

rate_down = @logic ? 0 : $d_TGFb;

}

Node TGFBR {

logic = ((TGFb | (HIF1 & TGFb)) & !MED12);

rate_up = @logic ? $u_TGFBR : 0;

rate_down = @logic ? 0 : $d_TGFBR;

}

Node TNFalpha {

logic = (TNFalpha);

rate_up = @logic ? $u_TNFalpha : 0;

rate_down = @logic ? 0 : $d_TNFalpha;

}

Node TSC1_2 {

logic = ((AMPK | p53 | HIF1) & !AKT & !ERK & !RAF & !p90RSK);

rate_up = @logic ? $u_TSC1_2 : 0;

rate_down = @logic ? 0 : $d_TSC1_2;

}

Node TWIST1 {

logic = (Snail | beta_catenin);

rate_up = @logic ? $u_TWIST1 : 0;

rate_down = @logic ? 0 : $d_TWIST1;

}

Node VEGF {

logic = (HIF1 & MYC_MAX);

rate_up = @logic ? $u_VEGF : 0;

rate_down = @logic ? 0 : $d_VEGF;

}

Node VHL {

logic = (!Hypoxia & !ROS);

rate_up = @logic ? $u_VHL : 0;

rate_down = @logic ? 0 : $d_VHL;

}

Node WNT {

logic = ((((ERG | AR_ERG) & GLI) | ERG | AR_ERG) & !p53);

rate_up = @logic ? $u_WNT : 0;

rate_down = @logic ? 0 : $d_WNT;

}

Node YWHAZ {

logic = (AR);

rate_up = @logic ? $u_YWHAZ : 0;

rate_down = @logic ? 0 : $d_YWHAZ;

}

Node ZBTB17 {

logic = (!MYC_MAX & AR);

rate_up = @logic ? $u_ZBTB17 : 0;

rate_down = @logic ? 0 : $d_ZBTB17;

}

**Example .cfg file**

$u_Acidosis=1;

$d_Acidosis=1;

$u_AKT=0.83536;

$d_AKT=1.19709;

$u_AMP_ATP=4.09511;

$d_AMP_ATP=0.24419;

$u_AMPK=1.30752;

$d_AMPK=0.76481;

$u_Androgen=1;

$d_Androgen=1;

$u_Angiogenesis=1;

$d_Angiogenesis=1;

$u_Apoptosis=1;

$d_Apoptosis=1;

$u_AR=2.73027;

$d_AR=0.36626;

$u_AR_ERG=1;

$d_AR_ERG=1;

$u_ATM=2.18312;

$d_ATM=0.45806;

$u_ATR=9.92017;

$d_ATR=0.1008;

$u_AXIN1=0.15753;

$d_AXIN1=6.348;

$u_BAD=0.05617;

$d_BAD=17.8031;

$u_Bak=0.44594;

$d_Bak=2.24245;

$u_BAX=0.57813;

$d_BAX=1.72971;

$u_BCL2=2.31731;

$d_BCL2=0.43153;

$u_Bcl_XL=7.13712;

$d_Bcl_XL=0.14011;

$u_beta_catenin=1.4429;

$d_beta_catenin=0.69305;

$u_BIRC5=0.07216;

$d_BIRC5=13.85809;

$u_BMP2=1;

$d_BMP2=1;

$u_BRCA1=0.57437;

$d_BRCA1=1.74104;

$u_BRCA2=0.82573;

$d_BRCA2=1.21105;

$u_Carcinogen=1;

$d_Carcinogen=1;

$u_Caspase3=1;

$d_Caspase3=1;

$u_Caspase8=3.68929;

$d_Caspase8=0.27105;

$u_Caspase9=0.40276;

$d_Caspase9=2.48287;

$u_CDH2=0.10074;

$d_CDH2=9.92654;

$u_cFLAR=2.59119;

$d_cFLAR=0.38592;

$u_CHK1_2=0.22021;

$d_CHK1_2=4.54112;

$u_COX4I2=0.16121;

$d_COX4I2=6.20309;

$u_CyclinB=0.50538;

$d_CyclinB=1.97871;

$u_CyclinD=0.76423;

$d_CyclinD=1.30851;

$u_CytoC=0.36821;

$d_CytoC=2.71584;

$u_APAF1=0.68619;

$d_APAF1=1.45732;

$u_DAXX=1.69245;

$d_DAXX=0.59086;

$u_DNA_Damage=1;

$d_DNA_Damage=1;

$u_DNA_Repair=1;

$d_DNA_Repair=1;

$u_Dsh=0.11729;

$d_Dsh=8.52588;

$u_E2F1=0.62905;

$d_E2F1=1.5897;

$u_E_cadherin=12.29395;

$d_E_cadherin=0.08134;

$u_eEF2=4.56862;

$d_eEF2=0.21888;

$u_eEF2K=1.54146;

$d_eEF2K=0.64874;

$u_EMT=1;

$d_EMT=1;

$u_EP300=8.46428;

$d_EP300=0.11814;

$u_ERG=100.0;

$d_ERG=0.01;

$u_ERK=0.80459;

$d_ERK=1.24287;

$u_ETS1=4.0028;

$d_ETS1=0.24983;

$u_ETV1=0.50943;

$d_ETV1=1.96298;

$u_EZH2=0.21244;

$d_EZH2=4.70721;

$u_FADD=0.12285;

$d_FADD=8.14001;

$u_FOS=3.38003;

$d_FOS=0.29586;

$u_JUN=0.38542;

$d_JUN=2.59457;

$u_FOXA1=2.24597;

$d_FOXA1=0.44524;

$u_FOXO=1.42832;

$d_FOXO=0.70012;

$u_EGF=3.56058;

$d_EGF=0.28085;

$u_EGFR=3.81647;

$d_EGFR=0.26202;

$u_FGF=0.15673;

$d_FGF=6.3804;

$u_FGFR3=0.12303;

$d_FGFR3=8.1281;

$u_FRS2=2.53061;

$d_FRS2=0.39516;

$u_GADD45=2.25563;

$d_GADD45=0.44334;

$u_GLI=2.85531;

$d_GLI=0.35022;

$u_GLUT1=0.60953;

$d_GLUT1=1.64061;

$u_Glycolysis=1;

$d_Glycolysis=1;

$u_GSH=0.42021;

$d_GSH=2.37976;

$u_GSK3=2.10525;

$d_GSK3=0.475;

$u_HIF1=3.16288;

$d_HIF1=0.31617;

$u_HSPs=0.31668;

$d_HSPs=3.15776;

$u_Hypermethylation=1;

$d_Hypermethylation=1;

$u_Hypoxia=1;

$d_Hypoxia=1;

$u_IDH1=0.54274;

$d_IDH1=1.8425;

$u_Invasion=1;

$d_Invasion=1;

$u_IKK=5.62168;

$d_IKK=0.17788;

$u_JNK=1.289;

$d_JNK=0.7758;

$u_Lactic_acid=2.30786;

$d_Lactic_acid=0.4333;

$u_LDHA=2.30786;

$d_LDHA=0.4333;

$u_MAP3K1_3=1.97475;

$d_MAP3K1_3=0.50639;

$u_MDM2=3.21016;

$d_MDM2=0.31151;

$u_MED12=5.11518;

$d_MED12=0.1955;

$u_MEK1_2=0.60079;

$d_MEK1_2=1.66448;

$u_Metastasis=1;

$d_Metastasis=1;

$u_Migration=1;

$d_Migration=1;

$u_mTORC1=1.52572;

$d_mTORC1=0.65543;

$u_mTORC2=1.46713;

$d_mTORC2=0.6816;

$u_MXI1=0.60156;

$d_MXI1=1.66234;

$u_MYC=3.51297;

$d_MYC=0.28466;

$u_MYC_MAX=2.10771;

$d_MYC_MAX=0.47445;

$u_NCOA3=2.36415;

$d_NCOA3=0.42299;

$u_NCOR1=0.35774;

$d_NCOR1=2.79533;

$u_NCOR2=4.45165;

$d_NCOR2=0.22464;

$u_NF1=6.13638;

$d_NF1=0.16296;

$u_NF_kB=0.96934;

$d_NF_kB=1.03163;

$u_NKX3_1=4.16047;

$d_NKX3_1=0.24036;

$u_Nutrients=1;

$d_Nutrients=1;

$u_p14ARF=0.83074;

$d_p14ARF=1.20375;

$u_p15=0.11023;

$d_p15=9.07194;

$u_p21=0.15109;

$d_p21=6.61857;

$u_p38=2.03731;

$d_p38=0.49084;

$u_p53=0.32579;

$d_p53=3.06946;

$u_p70S6kab=1.65289;

$d_p70S6kab=0.605;

$u_p90RSK=2.45014;

$d_p90RSK=0.40814;

$u_PDK1=1.48036;

$d_PDK1=0.67551;

$u_PHDs=0.43485;

$d_PHDs=2.29964;

$u_PI3K=1.91625;

$d_PI3K=0.52185;

$u_PIP3=1.60127;

$d_PIP3=0.6245;

$u_PKC=1.70874;

$d_PKC=0.58523;

$u_Proliferation=1;

$d_Proliferation=1;

$u_PTCH1=1.5434;

$d_PTCH1=0.64792;

$u_PTEN=1.24737;

$d_PTEN=0.80169;

$u_Quiescence=1;

$d_Quiescence=1;

$u_RAF=1.39718;

$d_RAF=0.71573;

$u_RAGS=0.17369;

$d_RAGS=5.75738;

$u_RAS=0.39058;

$d_RAS=2.56029;

$u_RB1=11.43462;

$d_RB1=0.08745;

$u_Rheb=0.18294;

$d_Rheb=5.46627;

$u_ROS=0.73357;

$d_ROS=1.3632;

$u_RTK=1.38402;

$d_RTK=0.72253;

$u_RUNX2=1.66538;

$d_RUNX2=0.60046;

$u_SHH=3.85043;

$d_SHH=0.25971;

$u_Slug=10.88394;

$d_Slug=0.09188;

$u_SMAD=4.66545;

$d_SMAD=0.21434;

$u_SMO=0.29637;

$d_SMO=3.37416;

$u_Snail=0.10973;

$d_Snail=9.11328;

$u_SPOP=5.27093;

$d_SPOP=0.18972;

$u_TAK1=3.36185;

$d_TAK1=0.29746;

$u_TCF=4.25645;

$d_TCF=0.23494;

$u_TERT=2.6768;

$d_TERT=0.37358;

$u_TGFb=0.31867;

$d_TGFb=3.13804;

$u_TGFBR=0.42193;

$d_TGFBR=2.37006;

$u_fused_event=1;

$d_fused_event=1;

$u_TNFalpha=1.05835;

$d_TNFalpha=0.94487;

$u_TSC1_2=1.18497;

$d_TSC1_2=0.8439;

$u_TWIST1=0.20413;

$d_TWIST1=4.89884;

$u_VEGF=0.57804;

$d_VEGF=1.72998;

$u_VHL=9.61299;

$d_VHL=0.10403;

$u_WNT=0.91257;

$d_WNT=1.09581;

$u_YWHAZ=0.25536;

$d_YWHAZ=3.91604;

$u_ZBTB17=0.31779;

$d_ZBTB17=3.14673;

discrete_time=0;

use_physrandgen=FALSE;

seed_pseudorandom=100;

//sample_count=500000;

sample_count=5000;

max_time=50;

//time_tick=0.01;

time_tick=0.1;

thread_count=1;

statdist_traj_count=100;

statdist_cluster_threshold=0.9;

display_traj=0;

statdist_similarity_cache_max_size=20000;

//artificialnodesistateshouldbenull

AKT.is_internal=TRUE;

AMPK.is_internal=TRUE;

AMP_ATP.is_internal=TRUE;

Androgen.is_internal=TRUE;

Angiogenesis.is_internal=TRUE;

APAF1.is_internal=TRUE;

Apoptosis.is_internal=FALSE;

AR.is_internal=TRUE;

AR_ERG.is_internal=TRUE;

ATM.is_internal=TRUE;

ATR.is_internal=TRUE;

AXIN1.is_internal=TRUE;

BAD.is_internal=TRUE;

Bak.is_internal=TRUE;

BAX.is_internal=TRUE;

BCL2.is_internal=TRUE;

Bcl_XL.is_internal=TRUE;

beta_catenin.is_internal=TRUE;

BIRC5.is_internal=TRUE;

BMP2.is_internal=TRUE;

BRCA1.is_internal=TRUE;

BRCA2.is_internal=TRUE;

Carcinogen.is_internal=TRUE;

Caspase3.is_internal=TRUE;

Caspase8.is_internal=TRUE;

Caspase9.is_internal=TRUE;

CDH2.is_internal=TRUE;

cFLAR.is_internal=TRUE;

CHK1_2.is_internal=TRUE;

COX4I2.is_internal=TRUE;

CyclinB.is_internal=TRUE;

CyclinD.is_internal=TRUE;

CytoC.is_internal=TRUE;

DAXX.is_internal=TRUE;

DNA_Damage.is_internal=TRUE;

DNA_Repair.is_internal=TRUE;

Dsh.is_internal=TRUE;

E2F1.is_internal=TRUE;

eEF2.is_internal=TRUE;

eEF2K.is_internal=TRUE;

EGF.is_internal=TRUE;

EGFR.is_internal=TRUE;

EMT.is_internal=TRUE;

EP300.is_internal=TRUE;

ERG.is_internal=TRUE;

ERK.is_internal=TRUE;

ETS1.is_internal=TRUE;

ETV1.is_internal=TRUE;

EZH2.is_internal=TRUE;

E_cadherin.is_internal=TRUE;

FADD.is_internal=TRUE;

FGF.is_internal=TRUE;

FGFR3.is_internal=TRUE;

FOS.is_internal=TRUE;

FOXA1.is_internal=TRUE;

FOXO.is_internal=TRUE;

FRS2.is_internal=TRUE;

fused_event.is_internal=TRUE;

GADD45.is_internal=TRUE;

GLI.is_internal=TRUE;

GLUT1.is_internal=TRUE;

Glycolysis.is_internal=TRUE;

GSH.is_internal=TRUE;

GSK3.is_internal=TRUE;

HIF1.is_internal=TRUE;

HSPs.is_internal=TRUE;

Hypermethylation.is_internal=TRUE;

Hypoxia.is_internal=TRUE;

IDH1.is_internal=TRUE;

IKK.is_internal=TRUE;

Invasion.is_internal=TRUE;

JNK.is_internal=TRUE;

JUN.is_internal=TRUE;

Lactic_acid.is_internal=TRUE;

LDHA.is_internal=TRUE;

MAP3K1_3.is_internal=TRUE;

MDM2.is_internal=TRUE;

MED12.is_internal=TRUE;

MEK1_2.is_internal=TRUE;

Metastasis.is_internal=TRUE;

Migration.is_internal=TRUE;

mTORC1.is_internal=TRUE;

mTORC2.is_internal=TRUE;

MXI1.is_internal=TRUE;

MYC.is_internal=TRUE;

MYC_MAX.is_internal=TRUE;

NCOA3.is_internal=TRUE;

NCOR1.is_internal=TRUE;

NCOR2.is_internal=TRUE;

NF1.is_internal=TRUE;

NF_kB.is_internal=TRUE;

NKX3_1.is_internal=TRUE;

Nutrients.is_internal=TRUE;

p14ARF.is_internal=TRUE;

p15.is_internal=TRUE;

p21.is_internal=TRUE;

p38.is_internal=TRUE;

p53.is_internal=TRUE;

p70S6kab.is_internal=TRUE;

p90RSK.is_internal=TRUE;

PDK1.is_internal=TRUE;

PHDs.is_internal=TRUE;

PI3K.is_internal=TRUE;

PIP3.is_internal=TRUE;

PKC.is_internal=TRUE;

Proliferation.is_internal=FALSE;

PTCH1.is_internal=TRUE;

PTEN.is_internal=TRUE;

Quiescence.is_internal=FALSE;

RAF.is_internal=TRUE;

RAGS.is_internal=TRUE;

RAS.is_internal=TRUE;

RB1.is_internal=TRUE;

Rheb.is_internal=TRUE;

ROS.is_internal=TRUE;

RTK.is_internal=TRUE;

RUNX2.is_internal=TRUE;

SHH.is_internal=TRUE;

Slug.is_internal=TRUE;

SMAD.is_internal=TRUE;

SMO.is_internal=TRUE;

Snail.is_internal=TRUE;

SPOP.is_internal=TRUE;

TAK1.is_internal=TRUE;

TCF.is_internal=TRUE;

TERT.is_internal=TRUE;

TGFb.is_internal=TRUE;

TGFBR.is_internal=TRUE;

TNFalpha.is_internal=TRUE;

TSC1_2.is_internal=TRUE;

TWIST1.is_internal=TRUE;

VEGF.is_internal=TRUE;

VHL.is_internal=TRUE;

WNT.is_internal=TRUE;

YWHAZ.is_internal=TRUE;

ZBTB17.is_internal=TRUE;

[Acidosis].istate=0.5[1],0.5[0];

[Androgen].istate=0.5[1],0.5[0];

[Carcinogen].istate=0.5[1],0.5[0];

[Hypoxia].istate=0.5[1],0.5[0];

[Nutrients].istate=0.5[1],0.5[0];

[AKT].istate=0.48047[1],0.51953[0];

[AMP_ATP].istate=0.65307[1],0.34692999999999996[0];

[AMPK].istate=0.52911[1],0.47089000000000003[0];

[APAF1].istate=0.45911[1],0.54089[0];

[AR].istate=0.60905[1],0.39095[0];

[ATM].istate=0.58477[1],0.41523[0];

[ATR].istate=0.74913[1],0.25087000000000004[0];

[AXIN1].istate=0.29934[1],0.7006600000000001[0];

[BAD].istate=0.18738[1],0.81262[0];

[Bak].istate=0.41232[1],0.58768[0];

[BAX].istate=0.44051[1],0.55949[0];

[Bcl_XL].istate=0.71338[1],0.28662[0];

[BCL2].istate=0.59125[1],0.40874999999999995[0];

[beta_catenin].istate=0.53981[1],0.46019[0];

[BIRC5].istate=0.21457[1],0.78543[0];

[BRCA1].istate=0.4398[1],0.5602[0];

[BRCA2].istate=0.47921[1],0.52079[0];

[Caspase8].istate=0.64174[1],0.35826[0];

[Caspase9].istate=0.40126[1],0.59874[0];

[CDH2].istate=0.2508[1],0.7492[0];

[cFLAR].istate=0.60337[1],0.39663000000000004[0];

[CHK1_2].istate=0.33571[1],0.66429[0];

[COX4I2].istate=0.30185[1],0.69815[0];

[CyclinB].istate=0.4259[1],0.5741[0];

[CyclinD].istate=0.47081[1],0.52919[0];

[CytoC].istate=0.39152[1],0.60848[0];

[DAXX].istate=0.55713[1],0.44287[0];

[Dsh].istate=0.26732[1],0.73268[0];

[E_cadherin].istate=0.77242[1],0.22758[0];

[E2F1].istate=0.44967[1],0.55033[0];

[eEF2].istate=0.66495[1],0.33504999999999996[0];

[eEF2K].istate=0.54698[1],0.45302[0];

[EGF].istate=0.63788[1],0.36212[0];

[EGFR].istate=0.64542[1],0.35458[0];

[EP300].istate=0.7319[1],0.2681[0];

[ERG].istate=1.0[1],0.0[0];

[ERK].istate=0.47639[1],0.52361[0];

[ETS1].istate=0.65059[1],0.34941[0];

[ETV1].istate=0.42677[1],0.57323[0];

[EZH2].istate=0.33181[1],0.6681900000000001[0];

[FADD].istate=0.27234[1],0.72766[0];

[FGF].istate=0.29879[1],0.70121[0];

[FGFR3].istate=0.27251[1],0.72749[0];

[FOS].istate=0.63223[1],0.36777000000000004[0];

[FOXA1].istate=0.58785[1],0.41215[0];

[FOXO].istate=0.53871[1],0.46129[0];

[FRS2].istate=0.60081[1],0.39919000000000004[0];

[GADD45].istate=0.58832[1],0.41168000000000005[0];

[GLI].istate=0.61391[1],0.38609000000000004[0];

[GLUT1].istate=0.44625[1],0.55375[0];

[GSH].istate=0.40587[1],0.59413[0];

[GSK3].istate=0.58083[1],0.41917000000000004[0];

[HIF1].istate=0.62502[1],0.37498[0];

[HSPs].istate=0.37515[1],0.62485[0];

[IDH1].istate=0.43365[1],0.56635[0];

[IKK].istate=0.68747[1],0.31253[0];

[JNK].istate=0.52756[1],0.47243999999999997[0];

[JUN].istate=0.39648[1],0.6035200000000001[0];

[Lactic_acid].istate=0.5908[1],0.4092[0];

[LDHA].istate=0.5908[1],0.4092[0];

[MAP3K1_3].istate=0.57388[1],0.42612000000000005[0];

[MDM2].istate=0.62663[1],0.37337[0];

[MED12].istate=0.67722[1],0.32277999999999996[0];

[MEK1_2].istate=0.44468[1],0.55532[0];

[ZBTB17].istate=0.37553[1],0.6244700000000001[0];

[mTORC1].istate=0.54587[1],0.45413000000000003[0];

[mTORC2].istate=0.54162[1],0.45838[0];

[MXI1].istate=0.44482[1],0.55518[0];

[MYC].istate=0.63642[1],0.36358[0];

[MYC_MAX].istate=0.58095[1],0.41905000000000003[0];

[NCOA3].istate=0.59342[1],0.40658000000000005[0];

[NCOR1].istate=0.38839[1],0.61161[0];

[NCOR2].istate=0.66213[1],0.33787[0];

[NF_kB].istate=0.49662[1],0.5033799999999999[0];

[NF1].istate=0.69698[1],0.30301999999999996[0];

[NKX3_1].istate=0.65479[1],0.34521[0];

[p14ARF].istate=0.47987[1],0.52013[0];

[p15].istate=0.26058[1],0.73942[0];

[p21].istate=0.29481[1],0.70519[0];

[p38].istate=0.57726[1],0.42274[0];

[p53].istate=0.37824[1],0.62176[0];

[p70S6kab].istate=0.55456[1],0.44543999999999995[0];

[p90RSK].istate=0.5973[1],0.40269999999999995[0];

[PDK1].istate=0.54259[1],0.45741[0];

[PHDs].istate=0.40959[1],0.59041[0];

[PI3K].istate=0.57061[1],0.42939000000000005[0];

[PIP3].istate=0.55112[1],0.44887999999999995[0];

[PKC].istate=0.55817[1],0.44182999999999995[0];

[PTCH1].istate=0.54712[1],0.45287999999999995[0];

[PTEN].istate=0.524[1],0.476[0];

[RAF].istate=0.53631[1],0.46369000000000005[0];

[RAGS].istate=0.30995[1],0.69005[0];

[RAS].istate=0.39793[1],0.60207[0];

[RB1].istate=0.76456[1],0.23543999999999998[0];

[Rheb].istate=0.31557[1],0.68443[0];

[ROS].istate=0.46636[1],0.53364[0];

[RTK].istate=0.53529[1],0.46470999999999996[0];

[RUNX2].istate=0.55538[1],0.44462[0];
